# Toxin-mediated tRNA^Trp^ depletion drives Trp-codon-dependent reprogramming in *Mycobacterium tuberculosis* while selectively enriching the PDIM biosynthetic machinery

**DOI:** 10.1371/journal.ppat.1014679

**Published:** 2026-10-12

**Authors:** Safreen Sain, Unnati Chauhan, Valdir C. Barth Jr, Eduardo A. Troian, Bruno L. Abbadi, Basanti Malakar, Robert N. Husson, Nancy A. Woychik

**Affiliations:** 1 Department of Biochemistry and Molecular Biology, Rutgers Health, Robert Wood Johnson Medical School, Piscataway, New Jersey, United States of America; 2 Division of Infecti ous Diseases, Department of Pediatrics, Boston Children’s Hospital, Harvard Medical School, Boston, Massachusetts, United States of America; University of Washington, UNITED STATES OF AMERICA

## Abstract

*Mycobacterium tuberculosis* (Mtb) must adapt to host-imposed stresses that limit its growth and ability to establish an infection. One important immune-regulated host defense mechanism involves competition between host and pathogen for tryptophan (Trp). Although Mtb is a Trp prototroph capable of synthesizing its own Trp, it normally acquires this amino acid from host macrophages because of the high energetic cost of its synthesis. However, interferon-γ-activated macrophages attempt to restrict Mtb growth by inducing indoleamine 2,3-dioxygenase 1 (IDO1) that degrades Trp. In response, Mtb senses and adapts to limitation of this essential amino acid by inducing its own Trp biosynthetic pathway, although the mechanisms coupling Trp limitation and compensatory Trp biosynthesis remain unknown. Here, we studied the Mtb VapC37 toxin and demonstrate that it arrests growth and is a highly selective tRNase that exclusively depletes tRNA^Trp^. Consequently, VapC37 triggers a state of perceived Trp starvation to upregulate production of Trp biosynthetic enzymes. Therefore, this toxin appears to link intracellular Trp availability—a well-established regulator of Mtb growth—to a codon-dependent adaptive response to increase the Trp concentration. VapC37 also broadly reprograms the Mtb transcriptome and translatome toward synthesis of mRNAs and proteins with low or no Trp codons. However, there is one striking exception: key enzymes in the phthiocerol dimycocerosate (PDIM) biosynthetic pathway were robustly synthesized despite being large and Trp-numerous, resulting in a > 2-fold increase in PDIM lipid production upon VapC37 expression. Thus, Mtb selectively preserves PDIM production under conditions of tRNA^Trp^ limitation. Finally, VapBC37 and PDIM transcripts are upregulated in Mtb cells subjected to nitrosative stress and upon Mtb infection of THP-1 macrophage-like cells. This might implicate early macrophage-associated stresses as activators of VapC37, possibly enabling this toxin to reallocate metabolic priorities toward Trp biosynthesis while selectively preserving PDIM production critical for Mtb early immune escape and virulence.

## Author summary

Tryptophan (Trp), the least abundant amino acid in *Escherichia coli*, is likely also limiting in *Mycobacterium tuberculosis* (Mtb) since intracellular Trp concentration acts as a growth regulating switch. Although Mtb is a prototroph capable of synthesizing its own Trp, this pathway is energetically costly. Macrophage-residing Mtb cells are therefore thought to exploit the host environment as an exogenous source of Trp. In response, the host innate immune system restricts bacterial growth through interferon-γ-induced degradation of Trp. Although Trp availability is essential to establish an infection in mice, it is unclear how Mtb senses and adapts to host-imposed Trp limitation. Here we report that the VapC37 toxin might help Mtb adapt to the Trp-restricted environment encountered during macrophage infection. VapC37-mediated tRNA^Trp^ inactivation mimics Trp starvation and triggers the Trp biosynthetic pathway that may counter host-imposed Trp stress while simultaneously remodeling Mtb physiology and upregulating the phthiocerol dimycocerosate (PDIM) lipid biosynthetic pathway essential for Mtb virulence. VapC37 and PDIM biosynthetic transcripts are upregulated during infection of THP-1 macrophage-like cells, and VapC37 transcripts are also induced by nitrosative stress. Together, these findings implicate VapC37 as a possible mediator that links growth arrest, Trp biosynthesis, metabolic adaptation, and preservation of virulence during infection.

## Introduction

Tuberculosis, caused by *Mycobacterium tuberculosis* (Mtb), is once again the leading cause of death by a single infectious agent [1]. This ancient pathogen encounters many host-imposed stresses during infection that are intended to restrict growth and threaten its survival. The unusually abundant type II toxin-antitoxin (TA) modules harbored in the Mtb genome likely contribute to stress survival, although the targets of most toxins remain undefined. Among the 67 Mtb type II TA systems in the Mtb genome [2], 50 belong to the VapBC (virulence-associated protein) family (VapB, antitoxin; VapC, toxin). All characterized bacterial VapC toxins are highly specific endoribonucleases that cleave their RNA targets at a single site recognized through a combination of RNA sequence and structure. Host-associated stresses are thought to regulate the dynamic interaction between a labile VapB antitoxin (which inhibits toxin activity) and its stable cognate VapC toxin. Release of the free toxin then allows it to recognize and cleave its RNA substrate. Therefore, precise identification of toxin RNA targets is essential for understanding the physiological consequences of VapC activation in Mtb.

Only six Mtb VapC toxins have been characterized in sufficient detail to permit precise identification of their RNA targets in Mtb cells. Five of these VapC toxins are highly specific tRNases that cleave a single site within the anticodon loop to inactivate a specific tRNA species. Three of the five are elongator tRNases: VapC4 targets tRNA^Cys^ [3], VapC11 targets tRNA^Gln^ [4], and VapC36 targets tRNA^Ser^ [5]. Two additional VapC toxins inactivate initiator tRNA^fMet^ [6]. Finally, Mtb VapC20 inactivates the ribosome by cleaving 23S rRNA at the highly conserved, essential sarcin-ricin loop (SRL) [7]; Mtb VapC26 was also reported to cleave the SRL when ectopically expressed in *Mycobacterium smegmatis* [8].

Here we used a multiomic approach to pinpoint tRNA^Trp^ as the sole target of the VapC37 toxin. Depletion of tRNA^Trp^ arrests growth and functionally mimics tryptophan (Trp) starvation, triggering upregulation of the Trp biosynthesis pathway. In parallel, VapC37 broadly reprograms the transcriptome and proteome away from Trp-numerous proteins while selectively preserving synthesis of the large, and highly Trp-numerous PpsA-E and Mas polyketide synthase outlier proteins required for phthiocerol dimycocerosate (PDIM) biosynthesis, resulting in increased PDIM lipid production. Finally, we show that *vapBC37* and PDIM biosynthetic transcripts are induced by nitrosative stress and during infection of THP-1 macrophage-like cells, suggesting a role for VapC37 within a host-relevant stress response that coordinates Trp homeostasis, metabolic adaptation, and preservation of virulence-associated lipid production.

## Results

### Expression of VapC37 leads to mycobacterial growth arrest

To simulate stress-induced toxin activation, we first expressed VapC37 in Mtb from the pristinamycin-inducible plasmid pMY769 [9], resulting in low level expression of free VapC37 (4.9-fold increase in toxin expression compared to the uninduced control; S1 Table). VapC37 expression inhibited Mtb growth in liquid cultures after two days followed by only a slight increase in OD_600_ up to day six (Fig 1A). We also expressed VapC37 in *M. smegmatis* from the anhydrotetracycline (ATc)-inducible pMC1s integrating plasmid specifically engineered by Ehrt et al. for low level mycobacterial expression [10]; here, growth quickly flatlined after VapC37 expression (Fig 1B). This rapid growth arrest in both Mtb and *M. smegmatis* is characteristic of an active VapC toxin and sets the stage for identifying its RNA target.

**Fig 1:**
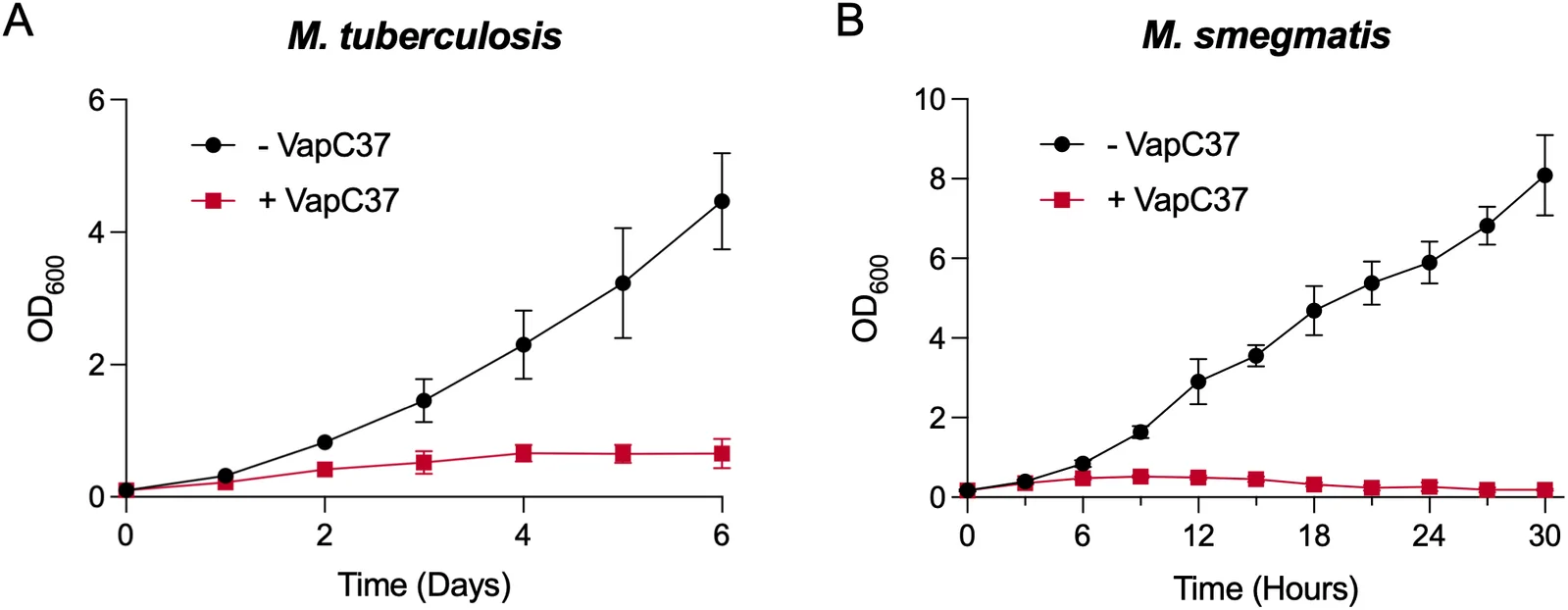
VapC37 expression induces growth arrest. Growth profiles in Mtb mc^2^6206 (A) and *M. smegmatis* mc^2^155 (B) demonstrate that VapC37 expression (+ VapC37; red boxes) results in reduced growth relative to the uninduced controls (- VapC37; black boxes) in both mycobacterial species. Error bars represent standard deviation, and cultures were grown in quadruplicate for each condition.

### VapC37 is a tRNase that inactivates the sole tRNA^Trp^

We applied our specialized 5’ end-specific RNA-seq method to identify the RNA target(s) of VapC37. We grew Mtb cells with and without induction of VapC37 expression, harvested total RNA, and prepared 5’-monophosphate end (5’-P) RNA-seq libraries (all Mtb VapC toxins generate a 5’-P upon RNA cleavage [3]). After sequencing and data analysis, we identified tRNA^TrpCCA^ as the RNA target of VapC37 (Fig 2A, S2 Table). Mtb has one tRNA species that services the only codon for Trp, UGG; therefore, going forward we refer to tRNA^TrpCCA^ as tRNA^Trp^. A cleavage ratio plot comparing the control versus toxin-expressing dataset revealed the position of cleavage in the center of the tRNA (Fig 2B). The precise VapC37 cleavage site is within the loop of the anticodon stem loop immediately after the CCA anticodon (Fig 2C), resulting in the generation of tRNA^Trp^ halves which are no longer functional for protein synthesis (Fig 2D).

**Fig 2:**
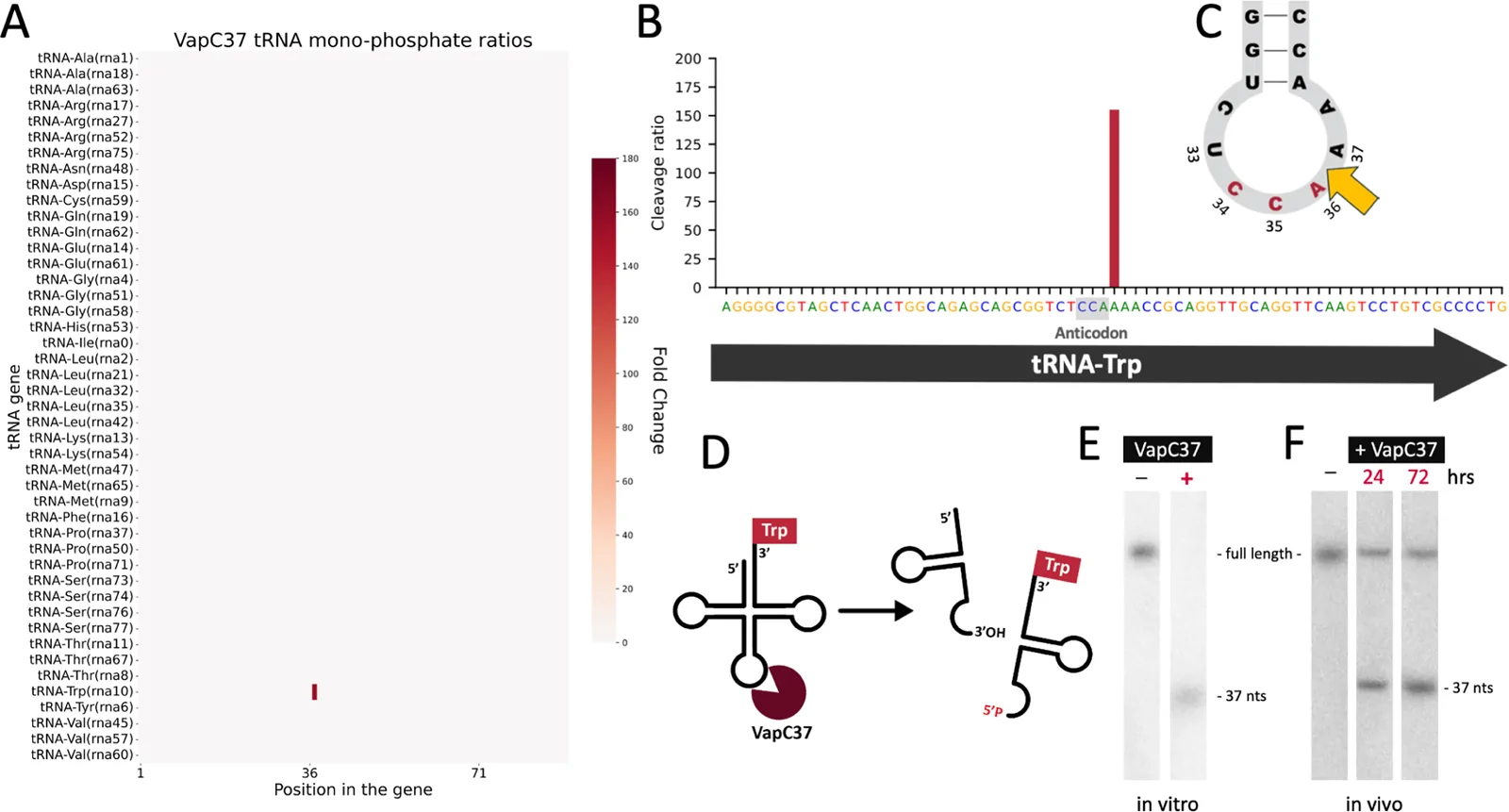
5’-P RNA-seq identifies tRNA^Trp^ as the sole target of VapC37. (A) Heatmap showing fold change ratios of 5’-P ends in +VapC37 expressed in Mtb mc^2^6206 cells relative to the uninduced control across all 45 Mtb tRNA species. (B) Bar graph showing fold change values of 5’-P ends within the tRNA^Trp^-encoding gene, *trpT*, in +VapC37 cells relative to uninduced cells. (C) The VapC37 cleavage site is located within the anticodon stem loop. (D) Diagram of tRNA-halves generated by VapC37-mediated cleavage of tRNA^Trp^. (E.F) Northern analysis validates cleavage of tRNA^Trp^ upon incubation of recombinant VapC37 with Mtb mc^2^6206 total RNA (E) and from RNA isolated from Mtb mc^2^6206 cells expressing VapC37 (F). Full-length tRNA^Trp^ and cleaved tRNA species are indicated.

To validate tRNA^Trp^ as the VapC37 target, we performed in vitro and in vivo northern analyses by hybridizing a labeled primer specific to the 3’ half of tRNA^Trp^ shown in Fig 2D. We first incubated recombinant VapC37 with total wild type Mtb RNA and observed complete cleavage of full length tRNA as indicated by hybridization to the 37 nt 3’ half of tRNA^Trp^ (Fig 2E). By contrast, hybridization of the same tRNA^Trp^-specific primer to RNA harvested from VapC37 expressing Mtb cells showed cleavage of tRNA^Trp^, but not to completion after 24 hrs or even 72 hrs (Fig 2F). Both 5’-P RNA-seq and northern analysis support tRNA^Trp^ as the only RNA target of VapC37, in alignment with other Mtb VapC toxins that also exhibit extraordinary target specificity for a single tRNA species [3,4,6].

### Trp codon-dependent remodeling of the transcriptome by VapC37

We have previously demonstrated that depletion of a specific tRNA isoacceptor has predictable downstream consequences for the transcriptome. Depletion of a single elongator tRNA species results in genome-wide ribosome stalling at the cognate codon(s) within mRNAs [3,11–13]. The transcripts bearing these paused ribosomes are cleaved by an unknown RNase, reducing their cellular abundance [3,11,13,14]. We quantified transcript levels in Mtb cells with or without VapC37 expression by RNA-seq (S3 Table, Worksheet 2). Among the 4098 transcripts detected 48 hrs following VapC37 expression, 1840 were statistically significant and differentially expressed (Fig 3A); within these, 880 transcripts were downregulated and 960 were upregulated. Each mRNA is displayed in a volcano plot showing its log_2_ fold change and statistical significance relative to the control, with Trp-codon content represented by circle diameter and color saturation, revealing that transcript abundance is inversely correlated with Trp codon content (Fig 3A). The corresponding box plot (Fig 3B) confirms this trend showing that Trp codons are significantly enriched in downregulated genes. Repressed transcripts exhibit higher median codon counts (median = 4) and show more high-end outliers with a wider distribution than the upregulated genes (median = 3). The plot also reveals a broader spread in Trp codon content with more high-value outliers in the downregulated group relative to the compressed distribution in the upregulated genes. Overall, there is a clear and statistically significant shift in support of systemic enrichment of Trp codons in genes with reduced expression (Fig 3B).

**Fig 3:**
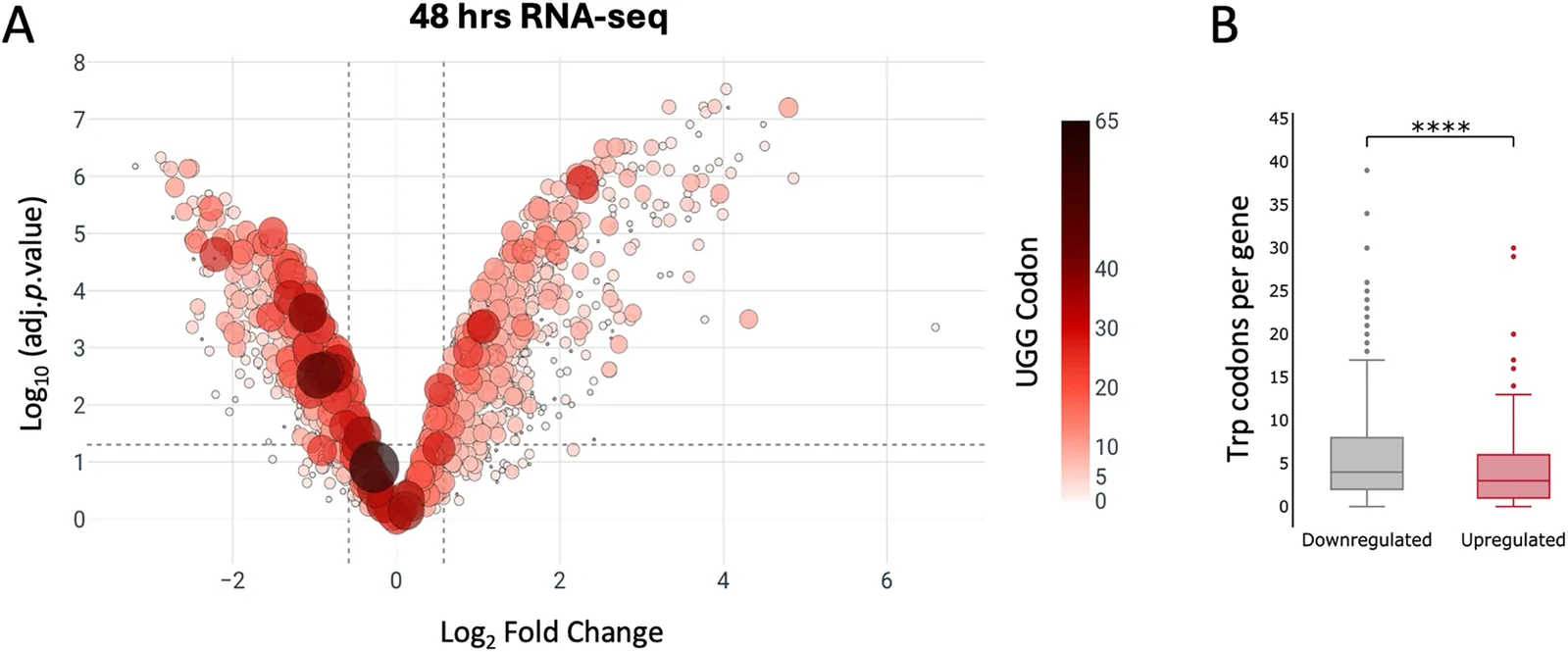
VapC37 mediates a Trp codon-dependent shift in transcript abundance. **(A)** Volcano plot showing relative transcript abundance (log_2_ fold change) in +VapC37 Mtb mc^2^6206 cells compared to the uninduced control 48 hrs following toxin induction. The horizontal gray dashed line denotes the statistical significance threshold (*q*-value ≤ 0.05), while the vertical gray dashed lines depict |log_2_ fold change| ≥ 0.58. Each circle represents an individual transcript; size and color correspond to the number of Trp codons. **(B)** Box-and-whisker plot comparing Trp codon content among statistically significant (*q*-value ≤ 0.05) downregulated (log_2_ fold change ≤ -1) and upregulated (log_2_ fold change ≥ 1) transcripts 48 hrs following VapC37 expression. Boxes represent the interquartile range (25^th^-75^th^ percentile), whiskers extend to values within 1.5x the interquartile range, and outliers are plotted as individual data points. Statistical significance was assessed using a two-sided Mann-Whitney U test *p*-value = 1.12 × 10^-7^.

### The translatome also displays Trp codon-dependent reprogramming after VapC37 expression

The impact of tRNA^Trp^ inactivation by VapC37 is expected to influence translation of proteins encoding Trp codons. Consequently, we assessed the effect of toxin expression on newly synthesized proteins following VapC37 induction using a nonradioactive in vivo metabolic labeling method called bioorthogonal noncanonical amino acid tagging (BONCAT) [15,16]. BONCAT incorporates azidohomoalanine (AHA), a variant of methionine that contains an azido group, into nascent proteins to enable their capture via Cu(I)-catalyzed azide–alkyne click chemistry onto an alkyne resin. Trypsin then facilitates the release of captured proteins from the resin which are subsequently identified and quantified by mass spectrometry. Among the 2205 newly synthesized proteins detected, 289 were statistically significant and differentially expressed; 208 were downregulated and 81 upregulated upon VapC37 induction (S1 Table). Detection of marginal levels of newly synthesized proteins in these growth-arrested cells is consistent with identification of low-level protein synthesis after TAMRA-azide fluorescence labeling of AHA-labeled Mtb proteins (S1 Fig).

In alignment with the effect of VapC37 on the transcriptome, proteins whose abundance decreased after toxin expression tend to have a higher Trp content than those whose synthesis was enhanced in Mtb (Fig 4) as well as *M. smegmatis* (S2 Fig, S4 Table). We initially included all but six strong outliers to best reveal the general trends in the volcano plot in Fig 4A. VapC37 expression leads to codon-dependent translational reprogramming, preferentially reducing synthesis of Trp-numerous proteins. The corresponding boxplot mirrored the general trends of VapC37 on transcripts consistent with Trp codon-dependent translational control (Fig 4B). Next, we added the six statistically significant outlier proteins to the volcano plot and boxplot (Fig 4C,D), revealing that 1) they were upregulated upon VapC37 expression despite high Trp content and 2) surprisingly they comprised enzymes in the PDIM biosynthesis pathway; all five polyketide synthases PpsA-E plus the mycocerosic acid synthase Mas (Fig 4C). Even with these six outlier proteins included, Trp content still differed significantly between down- and upregulated proteins (Fig 4D).

**Fig 4:**
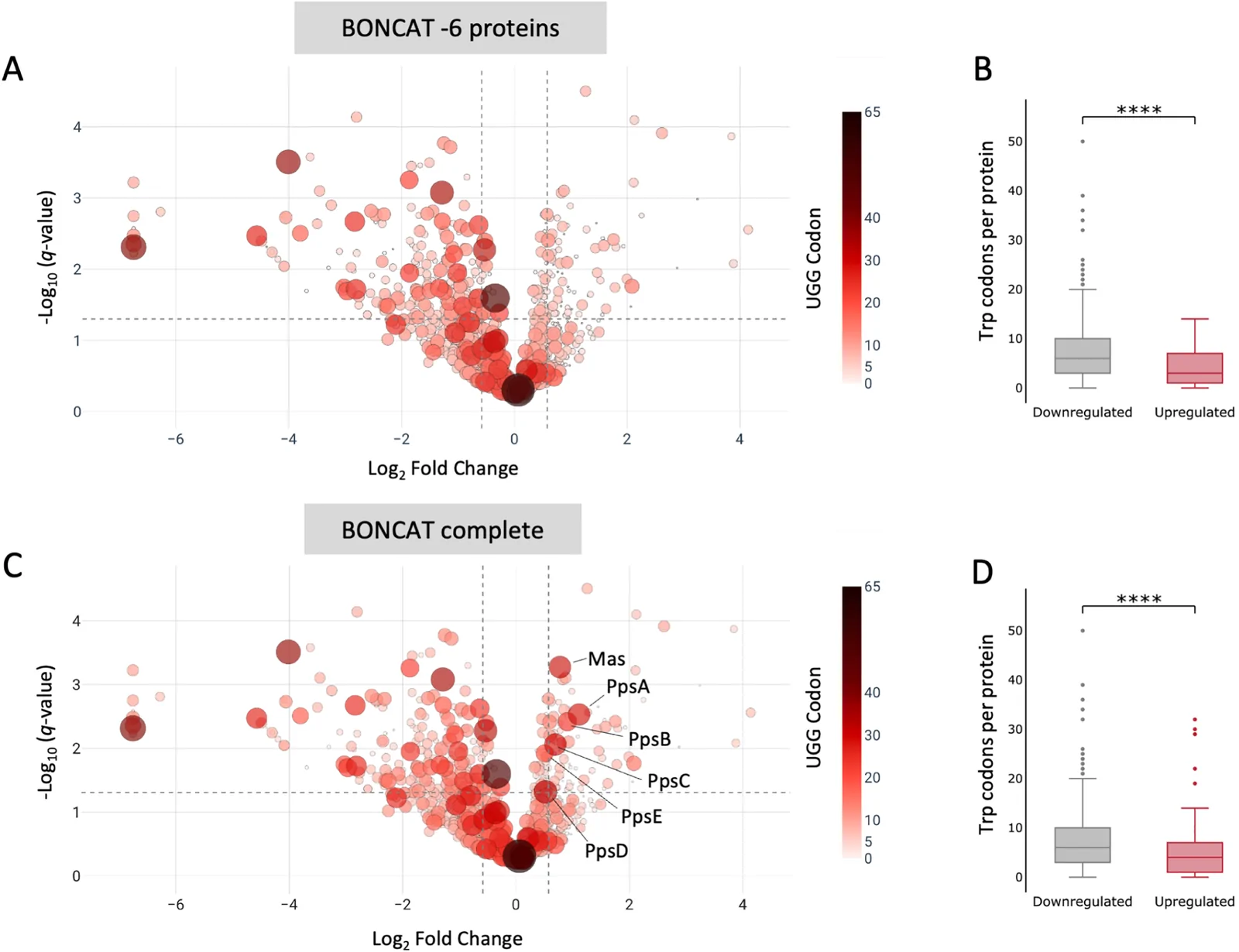
BONCAT mass spectrometry reveals Trp-codon dependent shifts in nascent protein synthesis following VapC37 expression. **(A and C)** Volcano plots showing log_2_ fold change ratios of newly synthesized proteins in VapC37-expressing in Mtb mc^2^6206 cells relative to uninduced control cells at 48 hrs. In panel A (“BONCAT -6 proteins”) the six major protein outliers in the PDIM biosynthesis pathway were removed to better visualize the global Trp codon-dependent shift in protein synthesis following VapC37 expression. Panel C (“BONCAT complete”) displays all proteins, including outlier proteins – PpsA, PpsB, PpsC, PpsD, PpsE, and Mas. Differential protein abundance cutoffs (± 1.5-fold change corresponding to a |log_2_ fold change| ≥ 0.58) and statistical significance (*q*-value ≤ 0.05) are represented by vertical and horizontal dashed lines, respectively. Each circle represents an individual protein; size and color correspond to the number of Trp codons. **(B and D)** Box and whisker plots illustrating Trp codon count of statistically significant (*q*-value ≤ 0.05) downregulated (log_2_ fold change < 0) and upregulated (log_2_ fold change > 0) proteins 48 hrs following VapC37 expression. Boxes represent the interquartile range (25^th^-75^th^ percentile), whiskers represent values within 1.5x the interquartile range, and outliers are plotted as individual datapoints. A two-sided Mann-Whitney U test was used to assess statistical significance; *p*-value = 1.01 × 10^-7^ (B) and *p*-value = 8.00 × 10^-6^ **(D)**.

Finally, we represented the 48 hr BONCAT and RNA-seq data as heat maps using the 233 mRNAs/proteins that were statistically significant (*q* ≤ 0.05) in both datasets (Fig 5A). In general, the effect of VapC37 was consistent on transcript and protein abundance—downregulated transcripts aligned with downregulated proteins (Fig 5A, shades of blue tones) and vice versa (Fig 5A, shades of red). We also plotted BONCAT log_2_ fold change versus RNA-seq log_2_ fold change for a qualitative representation of panel A (Fig 5B). This scatterplot generally reveals concordance between the two datasets—red in the upper right (79 up in both) and blue in the lower left (124 down in both). The Spearman’s rank correlation coefficient (ρ) = 0.63, indicating that the changes in newly synthesized protein abundance generally occur in corresponding directions across datasets. The gray discordant points are in the minority (30 total). The 21 points in the upper left discordant group (negative BONCAT/positive RNA-seq) may represent translational repression stemming from transcripts harboring stalled ribosomes at Trp codons that have not yet been rescued and recycled.

**Fig 5:**
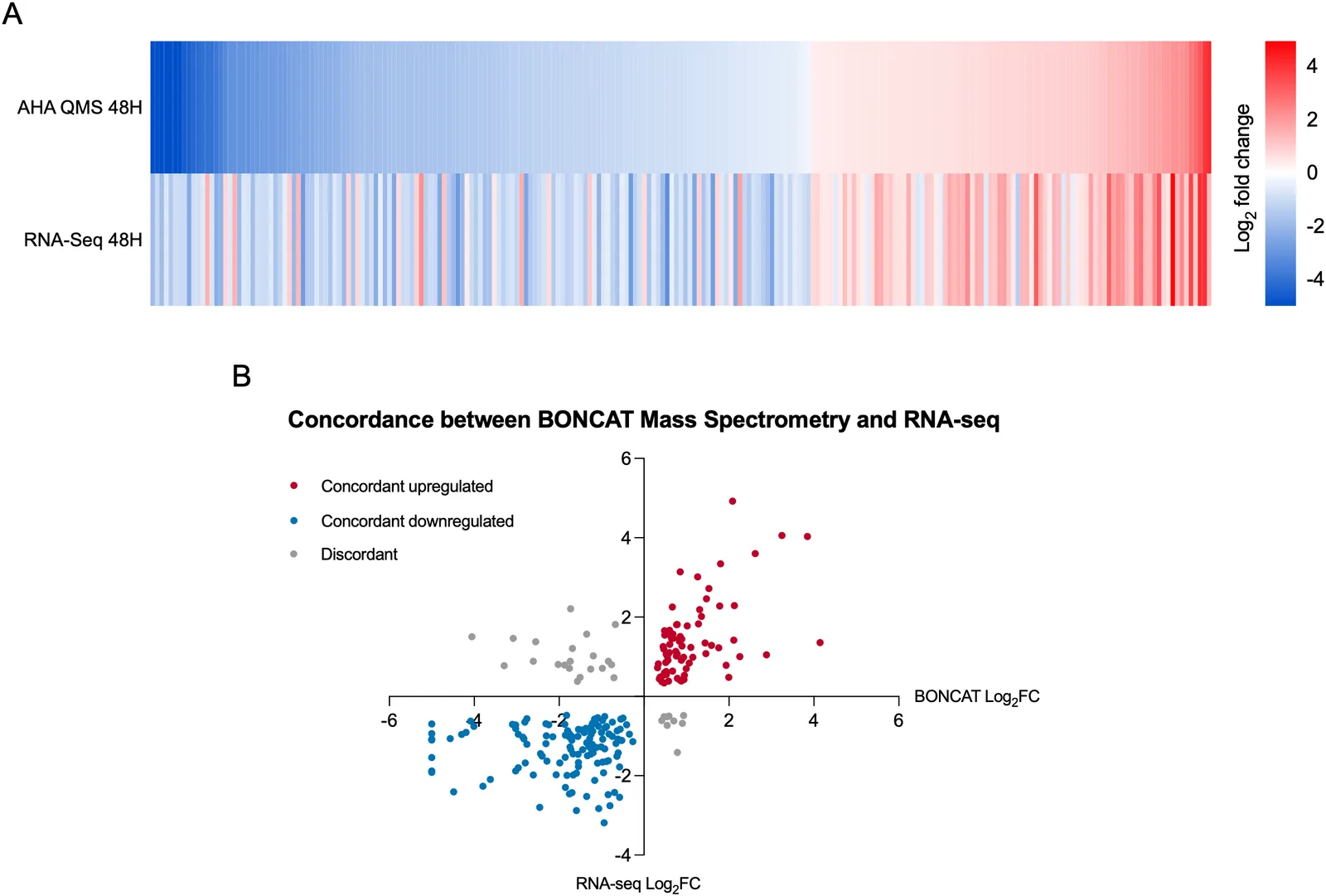
VapC37-induced changes in transcript abundance and nascent protein synthesis are broadly concordant. **(A)** Heatmap comparing log_2_ fold change values of transcripts at 48 hrs (RNA-seq) alongside corresponding changes in nascent protein synthesis at 48 hrs (BONCAT) following VapC37 expression in Mtb mc^2^6206 cells. Each colored line represents an individual gene or protein; RNA-seq transcripts are ordered relative to protein log_2_ fold change from BONCAT. **(B)** Scatterplot representation of panel A (discordant data points depicted in gray). The Spearman’s rank correlation coefficient (ρ) = 0.63. For panels A and B, the seven -16 log_2_ fold change downregulated points were adjusted to -5 to more closely reflected physiologically relevant changes.

### Large, Trp-numerous PDIM polyketide synthases PpsA-E and Mas escape the global trend following VapC37-mediaed tRNA^Trp^ depletion

To visualize how protein depletion related to protein size and Trp content, we ranked all BONCAT detected proteins by their log_2_ fold change—from most upregulated (blue) to most downregulated (orange)—and plotted amino acid count and Trp codon content for each protein (Fig 6). The PpsA-E and Mas polyketide synthases were immediately apparent as outliers as they were among the largest proteins detected (Fig 6A) and carried among the highest absolute numbers of Trp codons (Fig 6B). Yet all six were upregulated rather than depleted following VapC37 induction, placing them conspicuously among the upregulated proteins despite compositional features expected to sensitize them to Trp-dependent depletion. Because these six proteins were extreme with respect to both size and Trp content, we re-examined the distributions with PpsA-E and Mas removed (Fig 6C,D). In their absence, both protein size (Fig 6C) and Trp codon content (Fig 6D) were visibly skewed toward the downregulated proteins, indicating that, across the remainder of the translatome, larger and more Trp-rich proteins have a lower tendency to be synthesized when VapC37 is expressed. This qualitative relationship, and the disproportionate influence of the PpsA-E and Mas outliers on it, motivated the quantitative regression described in the following section.

**Fig 6:**
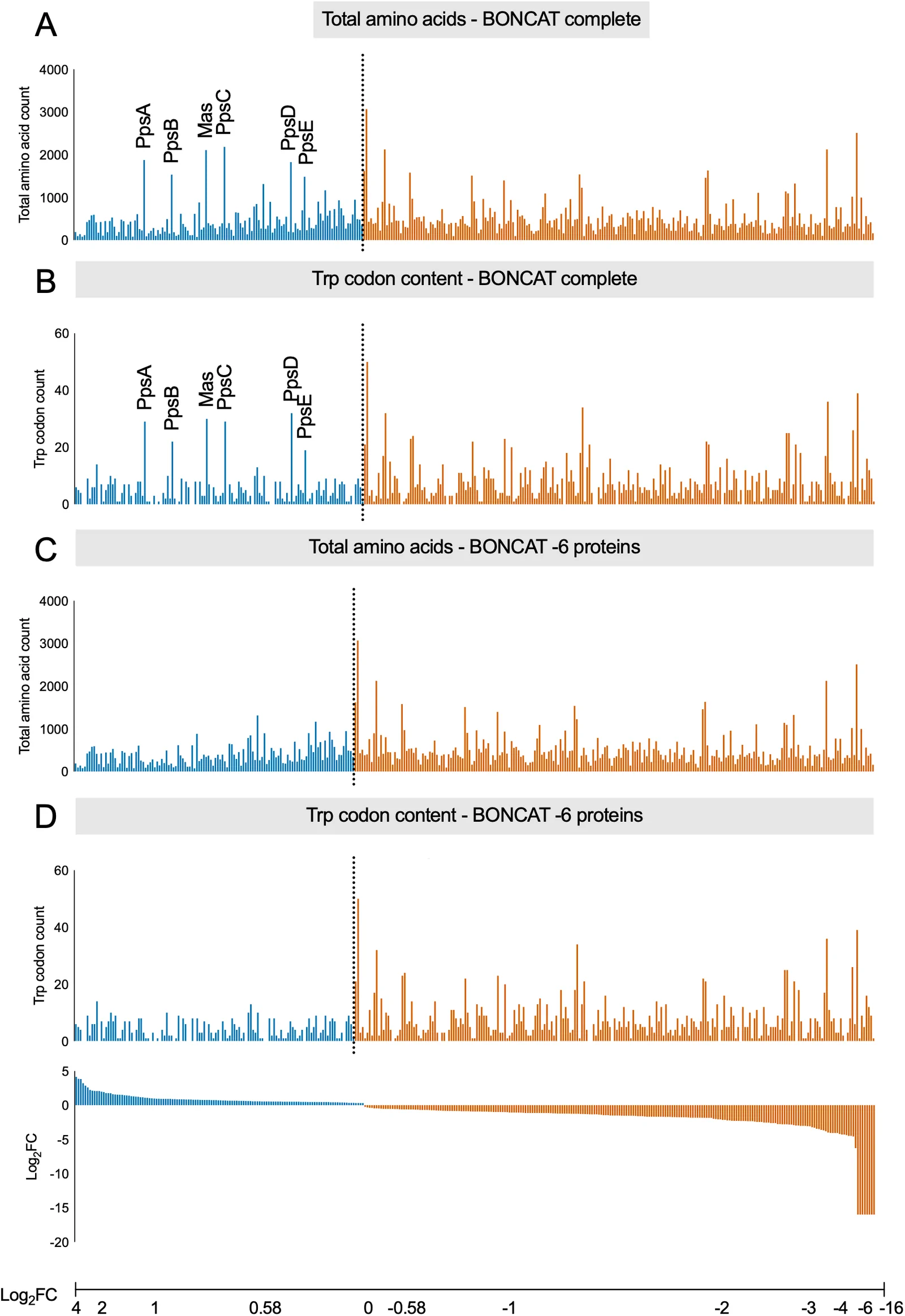
PpsA-E and Mas are exceptions to the size- and Trp-content trends associated with VapC37-mediated protein depletion. BONCAT-detected proteins were ranked by log_2_ fold change following VapC37 induction, from most upregulated (blue) to most downregulated (orange), and protein size and Trp content were plotted according to this ranking. **(A)** Total amino acid count (protein size) and (B) total Trp codon count for all significantly altered proteins detected by BONCAT (*q* ≤ 0.05; n = 349). PpsA-E and Mas are indicated and are among the largest and most Trp-numerous proteins detected despite being upregulated following VapC37 induction. **(C)** Total amino acid count and (D) total Trp codon count after exclusion of PpsA-E and Mas (n = 343) revealing the distribution of protein size and Trp content across the remaining BONCAT-detected proteins. The corresponding ranked BONCAT log_2_ fold change values are shown below. Multiple linear regression analyses evaluating the independent contributions of protein size and amino acid density are presented in Tables 1 and 2.

**Table 1:** Multiple linear regression analysis – BONCAT complete dataset.

	Size			Amino Acid Density				
**Amino Acid**	β*	**95% CI**	***p*-value**	**β**	**95% CI**	***p*-value**	**Model *p*-value**	**R** ^**2**^
Tryptophan	-0.00054	-0.0013 to 0.0002	0.1434	-49.68	-81.96 to -17.41	0.0026	0.0026	0.0337
Tyrosine	-0.00065	-0.0014 to 0.00009	0.0842	-12.63	-39.00 to 13.74	0.3467	0.1567	0.0107
Methionine	-0.00068	-0.0014 to 0.00007	0.0738	-10.62	-36.48 to 15.25	0.4200	0.1763	0.0100
Cysteine	-0.00063	-0.0014 to 0.0001	0.0951	1.801	-34.19 to 37.79	0.9217	0.2430	0.0081

*Trp: n=349, Tyr: n=349, Met: n=349, Cys: n=349.

**Table 2:** Multiple linear regression analysis – BONCAT-6 dataset.

	Size			Amino Acid Density				
**Amino Acid**	β*	**95% CI**	***p*-value**	**β**	**95% CI**	***p*-value**	**Model *p*-value**	**R** ^**2**^
Tryptophan	-0.001	-0.0019 to -0.0003	0.0125	-49.05	-81.28 to -16.81	0.003	0.0003	0.046
Tyrosine	-0.0011	-0.0020 to -0.0003	0.0067	-10.53	-36.92 to 15.86	0.4331	0.0195	0.023
Methionine	-0.0012	-0.0020 to -0.0004	0.0051	-11.57	-37.41 to 14.27	0.3789	0.018	0.023
Cysteine	-0.0011	-0.0020 to -0.0003	0.0073	0.4886	-35.48 to 36.45	0.9787	0.0265	0.021

*Trp: n=343, Tyr: n=343, Met: n=343, Cys: n=343.

To formally distinguish whether the depletion of specific proteins in the VapC37 BONCAT dataset was driven by their amino acid composition, we next performed multiple linear regression modeling of BONCAT log_2_ fold change against two predictors: total amino acid count (a measure of protein size) and the length-normalized density of a single amino acid using two datasets: all BONCAT proteins (“BONCAT complete”, Table 1) or the BONCAT dataset without PpsA-E and Mas (“BONCAT -6”, Table 2). Four such models were fit in each table, one each for Trp, Tyr, Met, and Cys (Trp compared to three other relatively rare amino acids in Mtb), across all statistically significant proteins detected by BONCAT mass spectrometry (*q* ≤ 0.05), regardless of the direction or magnitude of their fold change. Including total amino acid count in each model adjusts the density coefficient for protein length, isolating the contribution of amino acid density from that of size.

In the full BONCAT complete dataset, Trp density was the only significant predictor of depletion (β = -49.68, 95% CI -81.96 to -17.41; *p* = 0.0026), whereas the densities of Tyr, Met, and Cys were not (*p* = 0.35, 0.42. and 0.92, respectively). Protein size was not a significant predictor in any of the four models; no *p*-values were ≤ 0.05. The negative Trp density coefficient indicates that proteins with a higher proportion of Trp residues were underrepresented among newly synthesized proteins following VapC37 expression. Because protein size was included in the same models and was itself non-significant, this association reflects Trp density specifically rather than a tendency for larger proteins to contain more Trp. These results identify Trp density as the sole amino acid determinant of the depleted newly synthesized translatome, consistent with VapC37 acting specifically through tRNA^Trp^.

We noted, however, that the PpsA-E and Mas polyketide synthases represented extreme outliers with respect to both protein size and depletion direction (Figs 4, 6). To determine whether these proteins influenced the regression, we repeated the analysis with these six proteins removed (Table 2). Their exclusion markedly changed the contribution of protein size: whereas size was non-significant in the full dataset, it became a significant negative predictor of depletion in all four models. Trp density remained significant and essentially unchanged (β = -49.05, *p*-value = 0.003), and it remained the only amino acid density with a significant association; the densities of Tyr, Met, and Cys were non-significant in both analyses.

There are two conclusions from Tables 1 and 2. First, across the bulk of the translatome (i.e., everything but PpsA-E and Mas), VapC37-mediated depletion scales with both protein size and Trp density: larger proteins, and proteins with a higher proportion of Trp, are preferentially lost. Both dependencies are consistent with ribosome stalling upon tRNA^Trp^ depletion [3,14], which is expected to increase with the number of Trp codons a ribosome must traverse (a function of protein length) and with their local frequency (density). The size effect is amino acid nonspecific, as expected for a general property of protein length, whereas the density effect is specific to Trp, reinforcing that the depletion is driven by Trp content rather than amino acid composition generally.

Second, the size dependence was undetectable in the full dataset because it was masked by PpsA-E and Mas. These are among the largest proteins in the proteome and, by the size-dependent depletion that governs the rest of the proteome, would be expected to rank among the most depleted following VapC37 expression. Instead, they were elevated. Their exclusion unmasked the underlying size effect, and their behavior therefore represents a specific and pronounced exception to the size-dependent depletion operating across the proteome. This exception cannot be explained by amino acid composition and instead implicates a dedicated post-transcriptional mechanism that protects the synthesis of the PpsA-E/Mas machinery when VapC37 is active.

### Depletion of tRNA^Trp^ activates the Trp biosynthetic pathway

We then investigated how VapC37-mediated depletion of tRNA^Trp^ affects Trp metabolism. Host immune responses limit host-derived Trp to restrict bacterial growth [17–19], highlighting Trp availability as a key metabolic switch governing proliferation. Using mass spectrometry, the steady-state levels of all but two of the Trp biosynthetic enzymes were upregulated after VapC expression (Fig 7, S5 Table). The two exceptions, TrpG and TrpF, are the only enzymes that also function outside Trp biosynthesis. TrpG participates in both the synthesis of folate and Trp [20]. TrpF (*hisA*, Rv1603), is a bifunctional isomerase that participates in both Trp and His biosynthesis [17]. Unlike the other dedicated Trp biosynthetic enzymes, TrpG and TrpF/HisA levels might be constrained to maintain tight control of Trp pools because their increased enzyme abundance would disrupt flux of both pathways. Therefore, the coordinated increase in the abundance of much of the Trp biosynthetic machinery suggests that VapC37 expression mimics Trp starvation to upregulate Trp synthesis.

**Fig 7:**
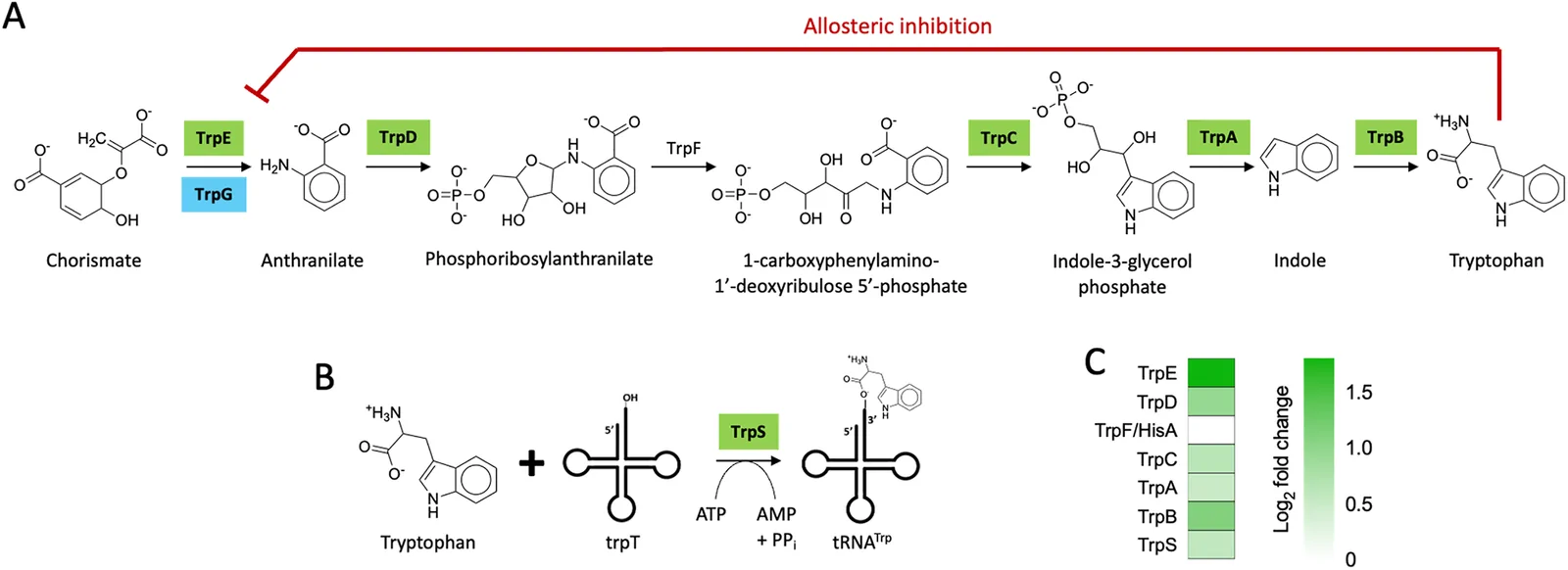
VapC37 expression upregulates enzymes in the Trp pathway. Quantitative mass spectrometry revealed that enzymes in the **(A)** Trp biosynthetic and (B) tRNA^Trp^ aminoacylation pathways increased upon VapC37 expression in Mtb mc^2^6206 cells. Enzymes boxed in green, steady-state; blue, BONCAT. TrpG was the only enzyme that was statistically significant in the BONCAT dataset (log_2_ fold change = 1.1, *q*-value 0.04). Panels A,B created with ChemDraw v25.0.2. **(C)** Heat map of the steady-state enzyme levels boxed in green in panels A and **B.**

### Functional annotation links VapC37 to synthesis of PDIMs

To better understand the broader effect of Trp codon-based reprogramming on Mtb physiology, we used the Database for Annotation, Visualization and Integrated Discovery (DAVID) functional annotation tool [21,22] to identify protein families enriched in our BONCAT dataset. Among the 81 upregulated proteins, DAVID analysis identified subsets of proteins that sorted into 14 annotation categories (Fig 8, S6 Table). However, proteins within different annotation categories were either identical (“ribosome” and “ribonucleoprotein”), related (“cytosolic small ribosomal subunit” and “cytosolic large ribosomal subunit”) or overlapping (“phthiocerol biosynthetic process” versus “fatty acid synthase activity”). Therefore, these 14 annotation categories represent only two discrete pathways: phthiocerol dimycocerosate (PDIM) biosynthesis and ribosome biosynthesis (Fig 8).

**Fig 8:**
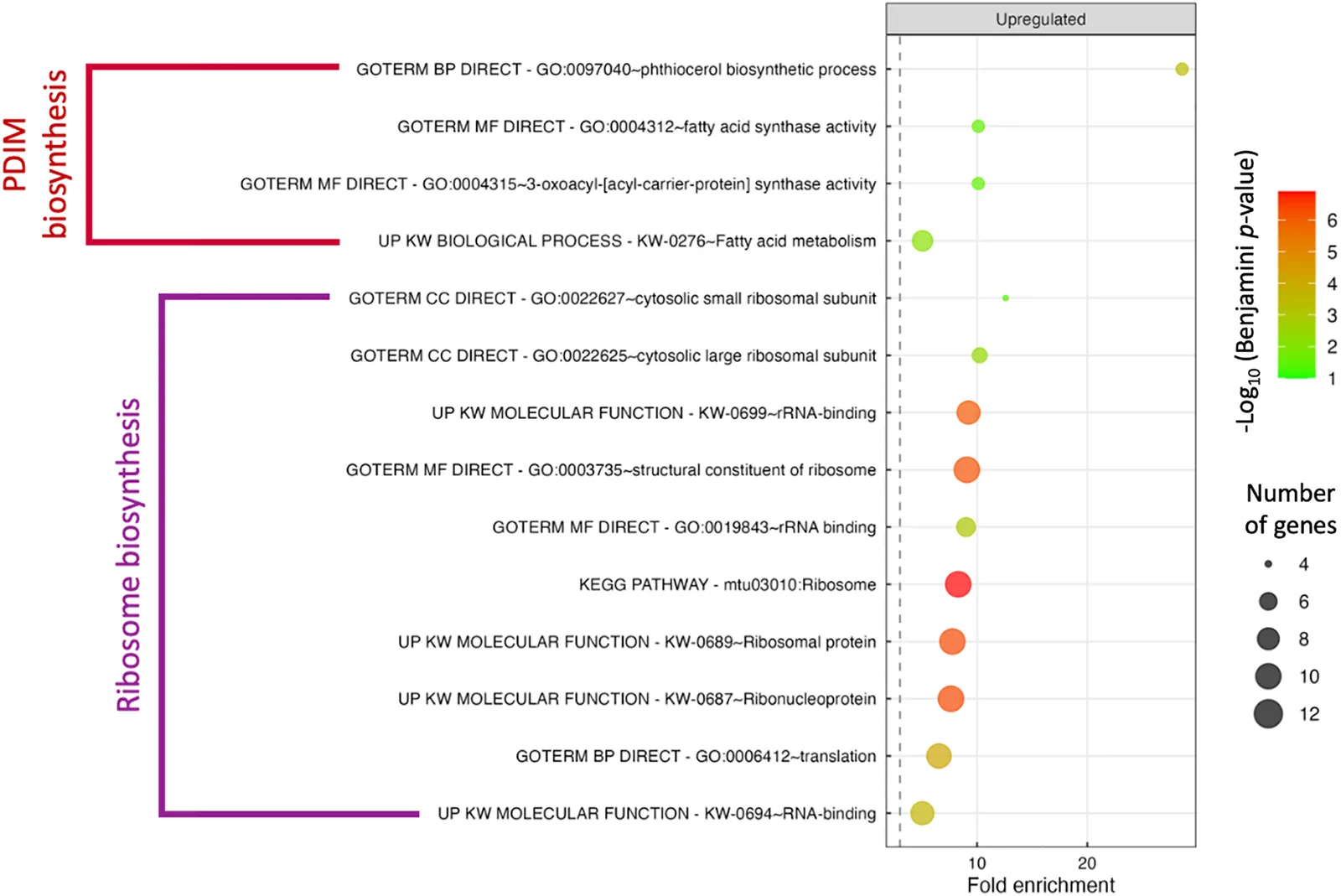
VapC37 enhances synthesis of ribosomal proteins and PDIM biosynthetic enzymes. DAVID Functional Annotation Tool analysis of the Mtb mc^2^6206 BONCAT mass spectrometry data identifies functional categories among significantly upregulated proteins (+ 1.5-fold change corresponding to a log_2_ fold change ≥ 0.58, *q*-value ≤ 0.05). Overlapping annotation categories were grouped and indicated by labeled brackets.

First, expression of 50 of the 57 Mtb ribosomal proteins increased in our BONCAT dataset because cells are likely in a state of ribosome stress (S1 Table, Worksheet 2). This is projected to be a consequence of depletion of tRNA^Trp^ by VapC37 that would generally lead to ribosome stalling at Trp codons, analogous to the genome-wide effects of another Mtb tRNase toxin that depletes a single Lys tRNA [14,23]. To remain viable, cells expressing VapC37 must maintain a threshold level of ribosomes by recycling those stalled at Trp codons or by synthesizing new ones. Mass spectrometry analysis of steady-state ribosomal protein levels for 55 of the 57 ribosomal proteins detected in this dataset revealed that VapC37 causes marked reductions in nearly all ribosomal proteins relative to the control (S5 Table). Thus, the upregulation of ribosomal proteins observed in our BONCAT dataset may offset the genome-wide shortage of free ribosomes.

Second, the enrichment of PDIM pathway enzymes uncovered upon DAVID analysis of the BONCAT dataset is consistent with the prominent PpsA-E and Mas datapoints among upregulated proteins in the volcano plot in Fig 4C. In fact, quantitative mass spectrometry measuring steady-state protein levels (rather than de novo synthesis) following VapC37 expression further supports the increased abundance of most enzymes and transporters within the contiguous 36-gene region of the Mtb genome encoding the PDIM pathway; many proteins exhibit striking 3–6 log_2_ fold changes relative to the control 24 and 48 hrs following VapC37 expression (Fig 9A-D). To evaluate whether the increased synthesis detected by BONCAT accounted for the elevated steady-state levels of PDIM proteins, we compared 48 hr log_2_ fold changes from BONCAT with those obtained from steady-state quantitative mass spectrometry for proteins quantified in both datasets (Fig 9E). All proteins increased in both analyses; however, the magnitude of the increase in steady-state protein levels was consistently greater than the increase in newly synthesized protein. BONCAT increases ranged from approximately 0.4 – 2.1 log_2_ fold change, whereas steady-state mass spectrometry increases reached approximately 1.2 – 6.0 log_2_ fold change (Fig 9E). Thus, enhanced synthesis alone may not fully account for the marked accumulation of these PDIM proteins, raising the possibility that protein stabilization also contributes to their elevated steady-state levels.

**Fig 9:**
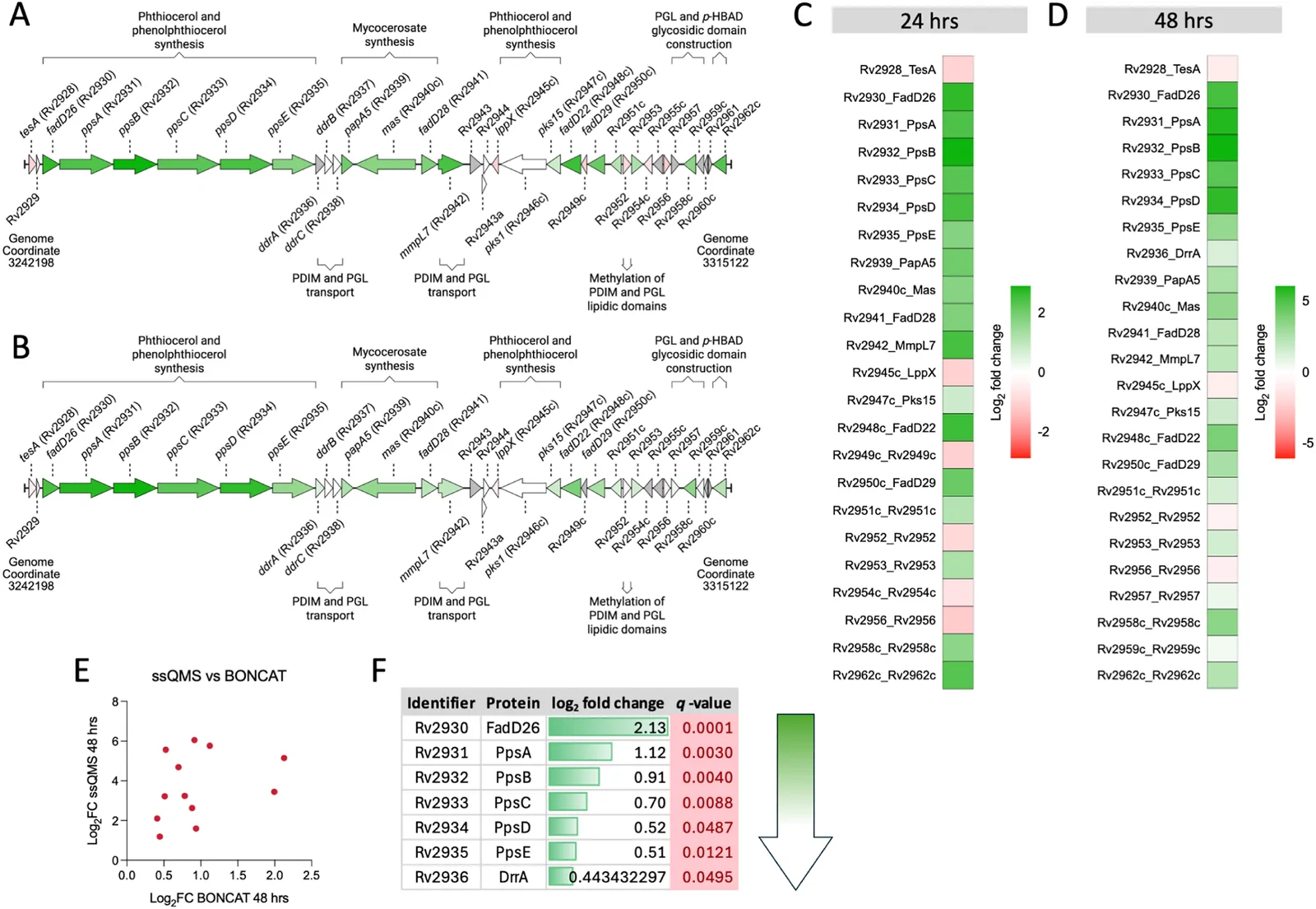
Proteomics reveals increased abundance of PDIM biosynthetic enzymes following VapC37 expression. **(A and B)** Mtb PDIM and PGL gene cluster, colored according to log_2_ fold change of corresponding proteins at 24 (A) and 48 hrs (B) after VapC37 induction in Mtb mc^2^6206 cells. Arrows represent individual genes and are drawn approximately to scale. Genes are colored based on the heatmap in panels C and D; those shown in white were not detected by mass spectrometry. **(C and D)** Heatmaps displaying log_2_ fold change values of statistically significant (*q-value* ≤ 0.05) PDIM and PGL biosynthetic identified by mass spectrometry 24 (C) and 48 hrs (D) after post-induction. **(E)** Scatterplot of statistically significant proteins (*q-value* ≤ 0.05) in 48 hr BONCAT and 48 hr steady-state quantitative mass spectrometry (ssQMS). **(F)** BONCAT data for the products of the *fadD26*- > *drrA* gene array within the Mtb PDIM gene cluster shown in panel **A.**

Finally, to determine whether the preferential synthesis and accumulation of PDIM pathway proteins observed in Fig 9 was accompanied by increased PDIM lipid levels, Mtb cells were labeled with [^14^C] propionic acid, the lipids extracted, and the PDIMs resolved by thin layer chromatography (TLC) (Fig 10A,B). In the unenhanced image (Fig 10A), PDIM levels were visibly greater following VapC37 expression. Contrast enhancement of the same TLC (Fig 10B) more clearly resolved both PDIM A and PDIM B and confirmed their increased accumulation in each of the four VapC37-expressing biological replicates. We then quantified PDIM levels in Mtb cells with or without VapC37 induction. Quantification of the PDIM bands revealed a 2.2-fold increase in total PDIM lipid production, supporting the proteomic data centered on this one pathway (Fig 10C). Thus, the selective preservation of PDIM biosynthetic machinery during tRNA^Trp^ depletion is accompanied by elevated levels of the mature lipid products.

**Fig 10:**
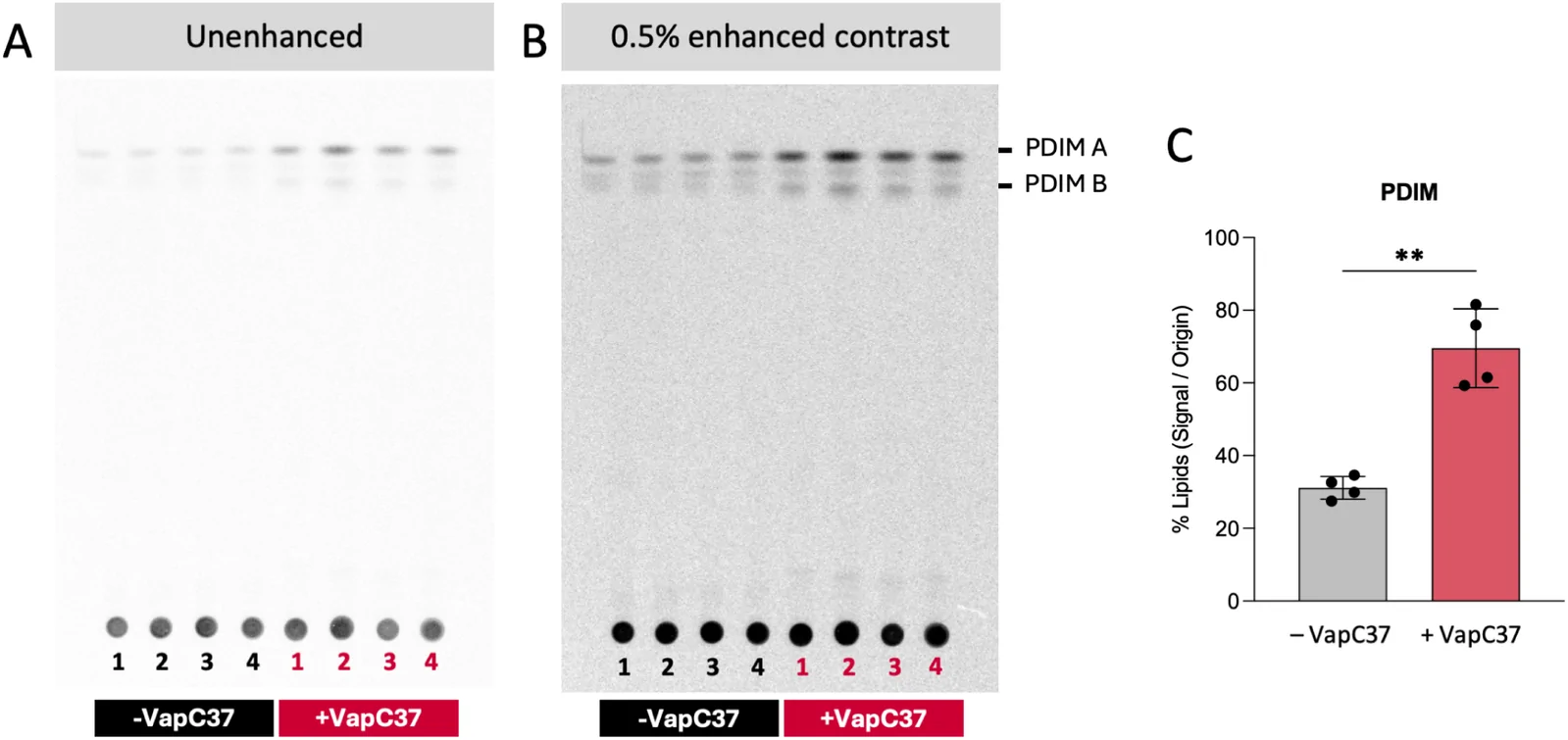
VapC37 enhances PDIM lipid levels. **(A and B)** Thin-layer chromatography of [^14^C] propionic acid labeled PDIMs from four replicates numbered at origin with (+VapC37) or without (-VapC37) toxin expression in Mtb mc^2^6206 cells using a 9:1 hexane: diethyl ether solvent system. Panel A shows the original TLC; panel B represents an enhanced-contrast version. **(C)** Quantification of the combination of PDIM A + B band intensities relative to the origin, expressed as % Lipids, demonstrates a statistically significant increase in final PDIM product following VapC37 expression. Error bars represent standard deviation; *p*-value = 0.01.

### Synthesis of FadD26, PpsA-E, and DrrA is coordinately regulated

The precise mechanism that selectively preserves production of this cell-wall associated lipid essential for Mtb virulence upon VapC37 induction is not clear. However, the BONCAT data for proteins encoded by the unidirectional *fadD26*- > *drrA* gene array within the Mtb PDIM gene cluster (Fig 9F) suggest coordinate regulation. Notably, synthesis of all proteins encoded by the *fadD26*-*ppsA-E-drrA* gene array was upregulated, with a striking progressive 5 - > 3’ decrease in newly synthesized protein levels, consistent with operon polarity. Yet the 24 and 48 hr RNA-seq datasets showed no clear evidence of transcriptional polarity across the *fadD26*-*ppsA-E-drrA* gene array; only *fadD26* exhibited increased transcription (log_2_ fold change ≥1; adjusted *p*-value ≤ 0.05) at both time points (1.8 and 2.3 log_2_ fold change 24 and 48 hrs post-VapC37 induction, respectively; S3 Table). The *fadD26* and *drrA* genes flank *ppsA-E* and encode proteins with distinct functions in PDIM biosynthesis and localization. FadD26 is required for PDIM biosynthesis (reviewed in [24]); it is a fatty acyl-AMP ligase that loads the activated fatty-acyl starter unit directly onto the polyketide synthase PpsA. PpsA-E then carry out successive elongation reactions that generate the phthiocerol backbone of PDIM. DrrA is the ATP-binding component of the DrrABC ABC transporter involved in exporting/localizing PDIM to the mycobacterial outer envelope, whereas DrrB and C comprise the membrane-associated components. Loss of *drrA, drrB, or drrC* prevents normal PDIM export to the outer envelope [25]. Thus, coordinate regulation of FadD26, PpsA-E, and DrrA would preserve the PDIM pathway from biosynthetic initiation and elongation through export/localization to the outer envelope.

### *vapBC37 and ppsA-E* expression increases in macrophage-associated stresses

Because limitation of host Trp availability occurs in interferon-γ-activated macrophages through induction of indoleamine 2,3-dioxygenase 1 (IDO1), and because we observed increased expression of the enzymes in the Mtb Trp biosynthetic pathway, VapC37 might contribute to adaptation to Trp stress during macrophage infection. To determine whether *vapC37* expression is induced during macrophage-associated stress, we analyzed published RNA-seq datasets derived from infected THP-1 macrophages separated into two populations, reduced and basal, gated by redox potential using a redox sensitive fluorescent probe [26,27]. Although the Singh group has previously separated THP-1 macrophages into populations enriched for reduced, basal, or oxidized mycothiol redox potential [28], Mishra et al. only reported RNA-seq data for reduced and basal populations [27]. The basal population reflects a baseline/intermediate redox state, whereas the reduced population represents an adaptive, drug-tolerant redox state. We evaluated expression of both *vapB* and *vapC* because they are transcribed in an operon; toxin activation occurs upon degradation of the antitoxin protein. The vapBC37 transcript increased in both intracellular populations relative to the in vitro control, with higher expression observed in the basal compared to the reduced redox population (Fig 11A). Notably, transcripts encoding the PDIM polyketide synthases PpsA-E were also elevated in both populations and generally showed greater enrichment in the basal redox population (Fig 11B).

**Fig 11:**
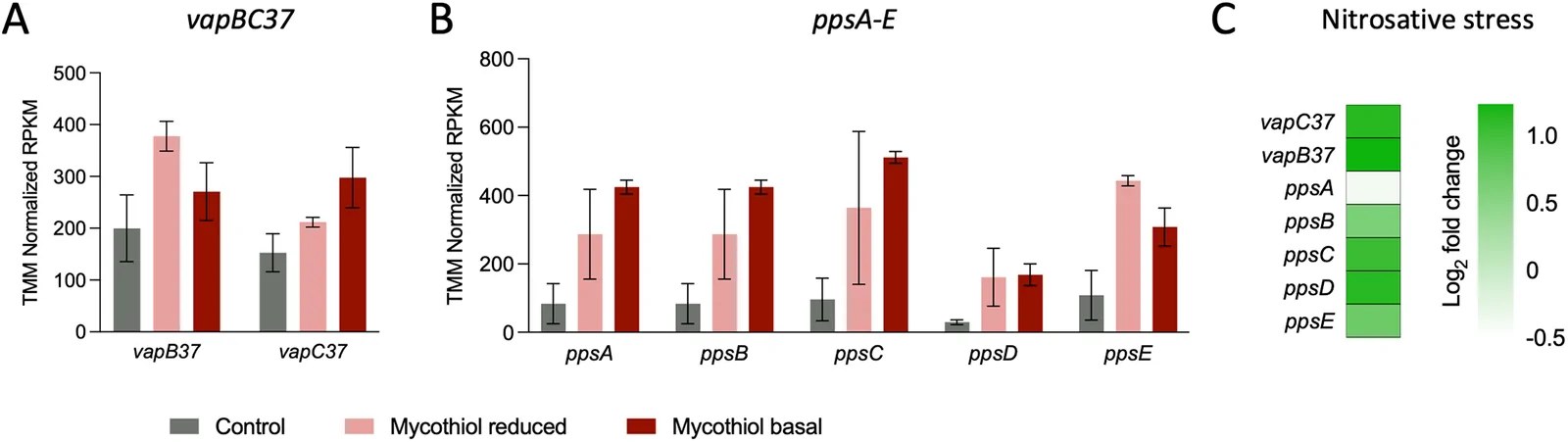
*vapBC37 and ppsA-E* transcripts are upregulated in Mtb in response to host-relevant stress conditions. (A and B) Transcript abundance of *vapBC37* (A) and *ppsA-*E (B) in Mtb grown in vitro (7H9 media) or following infection of THP-1 cells with Mtb H37Rv, sorted by mycothiol redox state [26,27]. (C) RNA-seq of Mtb H37Rv upon exposure to nitric oxide (DETA-NONOate) shows increased *vapBC37* and *ppsA-E* transcripts.

To determine if VapC37 responds to nitric oxide stress, we treated Mtb H37Rv cells with DETA-NONOate and performed RNA-seq analysis. Under these conditions, *vapBC* expression increased together with induction of nearly all *ppsA-E* polyketide synthase transcripts (Fig 11C). Because VapC37 expression elevates enzymes catalyzing Trp biosynthesis and tRNA charging and enhances PDIM lipid levels, these transcriptomic data implicate this toxin in contributing to a host-relevant stress response that couples macrophage-associated Trp limitation and nitrosative stress to remodeling of virulence-associated lipid metabolism. However, this association would be significantly strengthened by demonstration of tRNA^Trp^ cleavage under these conditions.

## Discussion

Mtb has adapted to survive a spectrum of stresses—nutrient limitation, oxidative and nitrosative stress, acidic pH—that collectively shape bacterial adaptation and persistence within the host following phagocytosis by permissive macrophages. Here, we used a multiomic approach to identify the RNA target of the Mtb VapC37 toxin, identify stresses that might trigger its activity, and obtain clues as to how this toxin might influence Mtb physiology and virulence. VapC37 toxin exclusively targets and inactivates the sole Trp tRNA, and this tRNA recognizes a single codon, UGG. Thus, tRNA^Trp^ cleavage by VapC37 removes the only decoding capacity for UGG codons, leaving Trp incorporation uniquely vulnerable. Indeed, we observed codon-based remodeling of the transcriptome and translatome generally favoring the production and translation of smaller mRNAs with fewer Trp codons over those with higher UGG codon content (Table 2).

Notably, despite being among the largest proteins in Mtb (ranging from ~1500–2200 amino acids), PpsA-E and Mas were preferentially synthesized upon VapC37 expression. The frequency of Trp in these PDIM biosynthetic enzymes is 1.5% (more precisely 1.458%), essentially identical to the overall frequency of Trp in the Mtb proteome (1.508%). Instead, their high absolute numbers of Trp residues, ranging from 19-32, simply track with protein size. In fact, multiple regression analysis in Tables 1 and 2 identified these proteins as clear outliers to the overall trend upon VapC37 expression, in which both protein size and Trp content negatively influence expression.

PpsA-E and Mas polyketide synthases are the core biosynthetic engines of PDIM production; without them, PDIM cannot be synthesized. PpsA-E synthesize the phthiocerol backbone, whereas Mas synthesizes mycocerosic acids that precede the final acylation steps required to produce the mature PDIM. Increased synthesis of these enzymes coincided with an over 2-fold increase in PDIM lipid levels despite genome-wide reprogramming away from Trp-numerous proteins. Therefore, identification of these protected outliers, together with many other enzymes and transporters encoded within the PDIM gene cluster (Fig 9), underscores the selective pressure for Mtb to prioritize production of this essential virulence factor (reviewed in [29–31]) under conditions expected to impair its synthesis.

The mechanism underlying protected translation of components of the PDIM machinery is not clear but appears to occur predominantly at the translational level since most of the corresponding transcripts did not increase in our RNA-seq datasets following VapC37 induction. Because global Trp codon-based reprogramming is initiated by VapC37, numerous uncharacterized physiological changes likely occur while the toxin is active. These changes may directly or indirectly contribute to the prioritized synthesis of the six polyketide synthases and the PDIM biosynthetic enzymes more broadly. Their preferential synthesis could involve formation of mRNA structures that enhance ribosome recruitment [32], alteration of ribosomal protein composition that favors translation of these transcripts [33], or preferential incorporation of alternate amino acids through adaptive mistranslation of the genetic code [34]. The increased steady-state levels of PDIM enzymes may also reflect increased stability of these proteins. Finally, we previously showed that a second tRNA-depleting TA toxin that cleaves tRNALysUUU reprograms Mtb to preserve both central metabolism and PDIM production when precursor lipids required for PDIM synthesis become limiting [23]. Therefore, preservation of PDIM production appears to be a recurring feature of toxin-mediated stress adaptation, underscoring the essential role of PDIMs in early immune evasion and virulence.

Trp is the least abundant amino acid in *Escherichia coli* [35] and likely in Mtb as well because intracellular Trp concentration is a known growth-regulating switch in Mtb [17–19]. Consistent with this observation, VapC37-mediated tRNA^Trp^ depletion leads to growth arrest and induces a functional Trp starvation response despite the absence of external nutrient limitation. This response is analogous to that which we described for Mtb VapC4 [3], a tRNA^Cys^-inactivating tRNase that mimics Cys starvation and upregulates the entire Cys biosynthesis pathway [3]. Cys feeds into the production of mycothiol, the glutathione analog in Mtb, because it provides the critical thiol (-SH) group that endows mycothiol with its redox activity. Mycothiol is central for Mtb survival in macrophages because it detoxifies reactive oxygen species, reactive nitrogen species, electrophiles, and toxic metals that can damage proteins or cause them to misfold [36].

Just as Cys is critical for Mtb survival, Trp availability is an important component of host nutritional immunity. Although Mtb is a Trp prototroph that encodes a complete Trp biosynthetic pathway, as illustrated in Fig 7, it is more energy efficient to acquire Trp from host cells. The indole aromatic structure of Trp makes it one of the most energetically expensive amino acids to synthesize in bacteria. Its synthesis diverts resources away from central carbon metabolism and consumes large amounts of reducing power. Consequently, Trp is among the rarest amino acids in proteins. Evolutionarily, Trp is incorporated sparingly in proteins and, when present, is not readily mutated. Indeed, when Trp (or Cys) residues are found to be mutated, they have the highest probability of causing human genetic diseases [37]. Thus, the high energetic cost and evolutionary conservation of Trp make its availability—and the cellular mechanisms that conserve its use—particularly important during Mtb adaptation to host-imposed nutritional stress.

The host innate immune system attempts to restrict Mtb growth through interferon-γ-induced degradation of Trp in macrophages by IDO1 [18,19]. Trp is required for Mtb survival in the phagosome and for pathogenesis since Trp auxotrophs are unable to establish an infection in mice [19]. Yet it remains unclear how Mtb senses and adapts to host-imposed Trp limitation. Here we show that the VapC37 toxin might help Mtb anticipate or adapt to the Trp-restricted environment encountered during macrophage infection. VapC37-mediated tRNA^Trp^ inactivation phenocopies Trp starvation, triggering the increased abundance of the enzymes in the Trp biosynthetic pathway that may counter host-imposed Trp stress. We also discovered that the very large, Trp-numerous polyketide synthases essential for PDIM biosynthesis are selectively synthesized when conditions are expected to impair their production. Therefore, the mechanisms ensuring their synthesis might be hardwired into the Mtb response to Trp insufficiency. In addition, *vapBC37* expression is increased in THP-1 macrophage-like cells and in response to nitrosative stress. Together, these findings may implicate VapC37 as a mediator linking growth arrest, Trp biosynthesis, metabolic adaptation, and preservation of virulence during infection, further supporting foundational studies that established Trp biosynthesis inhibitors as promising antitubercular targets [17,38].

## Materials and methods

### Strains, plasmids, and reagents

Plasmids utilized in this study were generated by amplifying *vapC37* (Rv2103c) from *Mycobacterium tuberculosis* H37Rv genomic DNA and cloning it into the anhydrotetracycline (ATc)-inducible vector pMC1s [9] or a pMY769-derived pristinamycin (PTM)-inducible vector [9].

To assess VapC37 toxicity in *Mycobacterium smegmatis* mc^2^155 (ATCC 700084) in liquid media, cultures were inoculated in 7H9 supplemented with 0.5% albumin, 0.2% dextrose, 0.085% NaCl, 0.05% Tween 80. Kanamycin (25 μg/mL) was added to the media to ensure plasmid selection. Cultures were incubated at 37°C with shaking at 170 rpm. When cultures reached an OD_600_ of 0.1, ATc (200 ng/mL; Takara Bio) was added to induce VapC37 expression. Following toxin expression, OD_600_ of cultures were measured every 3 hrs.

Except for the nitrosative stress RNA-seq performed with H37Rv, all Mtb studies were performed with *Mycobacterium tuberculosis mc*^*2*^*6206* (Δ*panCD* Δ*leuCD*), generously provided by the William Jacobs laboratory at Albert Einstein College of Medicine. Mtb was cultured in 7H9 media supplemented with 1 × oleic acid-albumin-dextrose catalase (OADC; BD Biosciences), 0.05% tyloxapol, 50 μg/mL pantothenic acid, 100 μg/mL leucine, and 100 μg/mL spectinomycin for plasmid selection. PTM (1 μg/mL; MilliporeSigma) was used to induce VapC37 expression once cultures reached an OD_600_ of 0.1. Cultures were incubated at 37°C with shaking at 170 rpm, and the OD_600_ was measured every 24 hrs.

For nitrosative stress treatment and RNA isolation, *Mycobacterium tuberculosis* H37Rv triplicate cultures were grown in 7H9 Middlebrook medium supplemented with 1X ADN (Albumin, Dextrose, and NaCl) until mid-log phase (OD_600_ 0.3-0.4) was reached. The cultures were then treated with DETA-NONOate (0.5 mM) for 1 hr. Cultures without any treatment were used as the control. Aliquots of 20 mL were centrifuged, and the pellets were resuspended in Tri Reagent (Zymo Research). Cells were lysed using three rounds of bead beating with 0.1 mm zirconia beads in a Mini-BeadBeater-16 (BioSpec Products) for 1 min at 4°C. RNA was extracted using the Direct-zol RNA Miniprep kit (Zymo Research) following the manufacturer instructions. RNA samples were treated with Turbo DNase (Thermo Fisher) to remove any residual genomic DNA and were purified using the RNA Clean and Concentrator Kit (Zymo Research).

### RNA isolation

To isolate RNA, ± VapC37 Mtb cells were cultured in triplicate, and 50 mL of each culture was collected at 24 and 48 hrs post-induction. Cell pellets were resuspended in 1 mL TRIzol and transferred to lysing tubes containing 200 μL of 0.1 mm zirconia/silica beads (BioSpec Products). Cells were lysed using a Precellys Evolution Touch Homogenizer (Bertin Technologies) for three 30-second cycles at 10,000 rpm, with 1-minute incubations on ice between each cycle. Lysed samples were centrifuged for 5 minutes at 4°C, and the supernatants were transferred to fresh tubes. RNA was subsequently purified from the supernatant using the Direct-zol RNA MiniPrep Plus Kit (Zymo Research). RNA samples were subjected to two consecutive rounds of genomic DNA digestion with TURBO DNase (Invitrogen) at 37°C for 30 minutes each. Samples underwent a second purification step using the RNA Clean and Concentrator Kit (Zymo Research). RNA concentrations were measured using a BioSpectrometer (Eppendorf).

### 5’ monophosphate RNA-seq

Libraries for 5’ monophosphate RNA-seq were prepared as previously described [3,4,6,14,39]. Raw sequencing reads from uninduced and induced samples were processed using a custom Bash pipeline. Adapter trimming and quality filtering were performed using Trim Galore! v0.6.10 (which utilizes Cutadapt) [40] with the following parameters: --phred33, minimum quality score of 20, minimum read length of 20 nucleotides, adapter stringency of 1, error rate of 0.1, and 6 nucleotides removed from the 5’ end. FastQC 0.12.1 [41] was used to assess the quality of trimmed reads. Reads were further trimmed to a length of 20 nucleotides using FASTX Toolkit v0.0.14 (Hannon Lab, GitHub). The processed reads were aligned to the Mtb H37Rv genome (GenBank accession AL123456.3) using Bowtie v1.3.1 [42] with the following parameters: -l 20 -n 0, allowing for zero mismatches. SAMtools v1.16.1 [43] was used to remove unmapped reads and remove alignments with a mapping quality score below 10. Custom Python scripts were used to generate strand-specific read counts corresponding to the 5’-P ends of aligned reads. Read counts were normalized to sequencing depth and expressed as reads per million (RPM) mapped reads. Genomic positions corresponding to 5’-P RNA ends were identified separately for each strand, and surrounding genomic sequences were extracted. A minimum cutoff of 1 RPM was applied to positions in the toxin-induced sample. 5’-P-end profiles were compared between uninduced and toxin-expressing samples by calculating the fold change in RPM-normalized read counts (induced/uninduced) at each genomic position. Positions exhibiting a ≥ 5-fold increase following toxin induction were retained as putative condition-dependent 5’-P RNA ends. These positions were annotated using *M*. *tuberculosis* H37Rv GFF3 genome annotations and CDS FASTA reference files. For Fig 2A,B we used a fold change and read count cutoff of ≥100 RPM.

### In vitro RNA cleavage assay with purified recombinant VapC37

To purify VapC37, *Escherichia coli* BL21 (DE3) was transformed with pET28a-*vapC37*. Cells were inoculated in M9 minimal media supplemented with 0.1% glycerol and 50 μg/mL kanamycin. Cultures were incubated at 37ºC until reaching exponential growth phase, at which protein expression was induced for 2 hrs upon addition of 1 mM isopropyl 1-thio-D-galacropyranoside (IPTG). Cells were harvested and lysed by sonication. VapC37 was purified from the cell lysate via nickel-nitrilotriacetic acid (Ni-NTA) affinity chromatography (Qiagen). 50 pmol of purified VapC37 was incubated with 4 μg total RNA from wild type Mtb, and cleavage reactions were incubated at 37ºC for 3 hrs. Samples were then analyzed by northern analysis as described below.

### Northern analysis of tRNAs

To detect in vitro and in vivo cleavage products generated by VapC37, we performed northern analysis using a DNA oligonucleotide specific to the 3’ end of Mtb tRNA^Trp^ (NWO2423, 5’-CAGGGGCGACAGGACTTGAACCTGCAA-3’). Primer specificity for tRNA^Trp^ was confirmed by hybridization of NWO2423 to a grid of all 45 Mtb in vitro synthesized tRNAs [44]. T4 polynucleotide kinase (New England Biolabs) and [γ-^32^P] ATP (Revvity) were used to radiolabel the 5’ end of the oligonucleotide. Total RNA (2 μg) was loaded onto a 15% polyacrylamide/7 M urea gel. Since these high percentage gels do not resolve 23S/16S or most mRNAs (they remain at the top of the gel), RNA quality and loading consistency was confirmed upon staining the gel with SYBR Gold (Thermo Fisher Scientific) prior to transfer. RNA was transferred to a nylon Hybond-N+ membrane (GE Healthcare) and incubated overnight at 54ºC with the radiolabeled probe in hybridization buffer (5 × SSC, 0.1% lauryl sarcosine, 0.02% SDS, 1 × Denhardt’s solution, and 25% formamide). Following hybridization, the membranes were washed twice for 15 minutes, first with 2 × SSC and 0.1% SDS, and then with 0.1 × SSC and 0.1% SDS. The membrane was exposed to a phosphorimager screen for 2 hrs and visualized using a Typhoon FLA 9500 imaging system (GE Healthcare).

### RNA-seq

rRNA was depleted from total RNA samples using an established protocol with biotinylated oligonucleotides specific to 23S, 16S, and 5S rRNAs [45]. Following rRNA depletion, RNA-seq libraries were prepared using the NEBNext Ultra II Directional RNA Library Prep Kit for Illumina (New England Biolabs).

Raw sequencing reads from ± VapC37 samples were processed using a custom Bash pipeline. Adapter trimming and quality filtering were performed using Trim Galore! v0.6.10 [40] with the following parameters: --phred33, minimum quality score of 20, minimum read length of 20 nucleotides, adapter stringency of 1, and error rate of 0.1. To remove residual rRNA reads, Bowtie2 v2.5.4 [46] was used to align trimmed reads to an rRNA reference index, and unaligned reads were retained for further analysis. The rRNA-depleted reads were then aligned to the Mtb H37Rv reference genome (GenBank accession AL123456.3) using Bowtie2. The alignment files were converted from SAM to BAM format, sorted, and indexed using SAMtools v1.16.1 [43]. Gene-level read counts were generated from the Mtb H37Rv GFF3 annotation using featureCounts [47] with strand-specific counting enabled (-s 2), gene features specified (-t gene), and gene identifiers assigned by ID attribute (-g ID). Differential gene expression analysis was performed in R using edgeR [48] and limma [49]. Counts were normalized using the trimmed mean of M-values method, transformed using voom [50], and analyzed using linear modeling with empirical Bayes moderation. Adjusted *p*-values were calculated using the Benjamini-Hochberg method.

### Labeling and detection of newly synthesized proteins

To assess changes in translation following VapC37 expression, Mtb cultures were grown in triplicate and induced at an OD_600_ of 0.1. After 24 hrs of toxin expression, ± VapC37 cultures were pulsed with 50 μM L-azidohomoalanine (AHA, Anaspec Inc.) for 12 hrs to label nascent proteins. Following labeling, 50 mL cultures were harvested, and cell pellets were resuspended with lysis buffer (2% CHAPS, 8 M Urea, pH 9) supplemented with cOmplete Mini EDTA-free protease inhibitor (MilliporeSigma) and Benzonase Endonuclease (MilliporeSigma). Cells were lysed via bead beating as described for RNA isolation. AHA-labeled proteins were precipitated via methanol-chloroform extraction and subsequently conjugated to the alkyne-containing fluorophore tetramethylrhodamine (TAMRA) using the Click-iT Protein Reaction Buffer Kit (Thermo Fisher Scientific). 7 μg of TAMRA-labeled protein from each sample were resolved on an SDS-PAGE gel. The gel was imaged using a Typhoon FLA 9500 imaging system (GE Healthcare). To verify equal protein loading, the gel was stained using a silver staining kit (Thermo Fisher Scientific).

### Proteomics

To investigate the VapC37-mediated changes in nascent protein synthesis in *M. smegmatis* and Mtb, ± VapC37 expressing cells were grown in triplicate. Upon reaching OD_600_ 0.1 for *M. smegmatis* cells or  40 hrs of induction in Mtb, AHA (Anaspec Inc.) was added to cells to 50 μM for 3 or 8 hrs, respectively. Cells were lysed by bead beating in lysis buffer (8 M urea, 200 mM Tris pH 8, 4% CHAPS, 1 M NaCl) and AHA-labeled proteins were isolated using the Click-It Protein Enrichment Kit (Thermo Fisher Scientific) as described in the manufacturer’s protocol. Column-bound proteins were digested using trypsin and analyzed using an Orbitrap Tribrid mass spectrometer coupled to a nanoflow LC system (Thermo Fisher Scientific) as previously described [14]. Differential protein abundance is presented as log_2_ fold change ratios of induced versus uninduced samples, and *q*-values were determined using the fdrtool package of Strimmer [51].

We additionally performed mass spectrometry to quantify total protein levels in uninduced and VapC37 expressing cells. Mtb ± VapC37 was grown in quadruplicate, and cells were harvested 24 and 48 hrs post-induction with PTM. Following bead beating of cells resuspended in TRIzol, we used a modified trizol-chloroform extraction protocol based on Hummon et al. to isolate proteins [52]. Proteins were digested with trypsin and the resulting peptides were analyzed by nanoLC-MS/MS using an Orbitrap Eclipse Tribrid Mass Spectrometer (Thermo Fisher Scientific), as described in Barth et al. [14]. Data were collected in data-independent acquisition (DIA) mode. The raw LC-MS data files were then converted to mzML format using MSConvert (ProteoWizard v3.0.25343) [53].

DIA-NN v1.9 [54] was used to generate a spectral library based on the Mtb H37Rv FASTA database using deep learning-based prediction of fragment ion spectra, retention times, and ion mobility. Peptide settings included a length range of 7–30 amino acids, precursor charge states of 2–4, precursor m/z range of 400–1200, and fragment ion m/z range of 200 – 1800. Smart profiling and peptidoform analysis were enabled. DIA datasets were searched against the generated spectral library and FASTA database using identical parameters. The DIA-NN output file was filtered to retain hits with *q*-values and posterior error probabilities (PEP) ≤ 0.01. Protein group intensities (PG.MaxLFQ) were extracted and organized into a matrix. The resulting dataset was imported into Perseus v2.1.1.0 [55]. Protein intensity values were log_2_ transformed, and proteins with at least three valid values within one sample group and at least four valid values in all samples were retained. Missing values were imputed from a normal distribution using Perseus default settings. Statistical significance was determined using a two-tailed, non-paired Student’s *t*-test and a permutation corrected FDR of 5%.

### Radioactive labeling and detection of PDIMs

To assess PDIM biosynthesis, uninduced and VapC37 expressing Mtb cultures were grown in quadruplicate. After 24 hrs of induction with 1 μg/mL PTM, 20 mL of ± VapC37 cultures were labeled with 0.4 µCi/mL propionic acid [1-^14^C] sodium salt (American Radiolabeled Chemicals, Inc.) in 50 mL conical tubes. Labeled cells were washed twice with water and pelleted 48 hrs after labeling. Lipids were isolated using a chloroform:methanol (2:1) extraction described previously [22]. To resolve PDIMs via thin layer chromatography, lipids were resuspended in chloroform:methanol (1:1) to reach a final volume of 1,000 counts per minute (CPM) per µL. For each sample, 10,000 CPM (10 μL) of lipids were spotted onto a 400 cm^2^ HPTLC silica gel sheet (MilliporeSigma) and resolved using hexane: diethyl ether (9:1). The TLC sheet was then exposed to a phosphorimager screen for two days and imaged using a Typhoon FLA 9500 imaging system (GE Healthcare). ImageJ 1.53K was used to quantify PDIM and origin intensities. To calculate relative PDIM intensity of each uninduced and induced sample, the intensities of PDIM A and PDIM B bands were divided by the intensity of the sample origin. The intensities of both PDIM bands were then summed, and mean PDIM band intensity was calculated for the uninduced and induced groups. Fold change increase in PDIMs was calculated by dividing the mean PDIM band intensity of VapC37 induced replicates by the mean PDIM band intensity of VapC37 uninduced replicates.

### Statistical analyses

GraphPad Prism software 10.1.0 was used for statistical analyses of growth profiles.

## Supporting information

S1 Fig VapC37 partially inhibits protein synthesis in Mtb.  ± VapC37 Mtb mc^2^6206 cells were pulsed with the methionine mimetic AHA to label newly synthesized proteins, which were then conjugated to TAMRA for detection by SDS-PAGE.
(TIF)

S2 Fig Expression of Mtb VapC37 in *M. smegmatis* mc^2^155 results in a Trp codon-dependent shift in newly synthesized proteins using BONCAT. Protein abundance cutoffs (± 1.5-fold change corresponding to a |log_2_ fold change| ≥ 0.58) and statistical significance threshold (*q*-value ≤ 0.05) are represented by vertical and horizontal dashed lines, respectively. Each circle represents an individual protein; size and color correspond to the number of Trp codons.
(TIF)

S3 Fig Raw images. (PDF)

S1 Table VapC37 BONCAT dataset. Tab 1: VapC37 BONCAT dataset. Tab 2: VapC37 BONCAT dataset - ribosomal proteins.
(XLSX)

S2 Table 5’-P RNA-seq dataset. (XLSX)

S3 Table VapC37 RNA-seq dataset. Tab 1: VapC37 RNA-seq dataset – 24 hr. Tab 2: VapC37 RNA-seq dataset – 48 hr.
(XLSX)

S4 Table *M. smegmatis* VapC37 BONCAT dataset. (XLSX)

S5 Table VapC37 proteomics dataset. Tab 1: VapC37 proteomics dataset – 24 hr. Tab 2: VapC37 proteomics dataset – 48 hr.
(XLSX)

S6 Table VapC37 DAVID output. (XLSX)

S7 Table *M. tuberculosis* H37Rv DETA-NONOate treatment dataset – 1 hr. (XLSX)
