# Supplementary material for: Toxin-mediated tRNATrp depletion drives Trp-codon-dependent reprogramming in Mycobacterium tuberculosis while selectively enriching the PDIM biosynthetic machinery
Source: PLoS Pathog. 2026 Oct 12;22(10):e1014679. doi: 10.1371/journal.ppat.1014679 (PMC13657306; doi:10.1371/journal.ppat.1014679)

Figure 2E

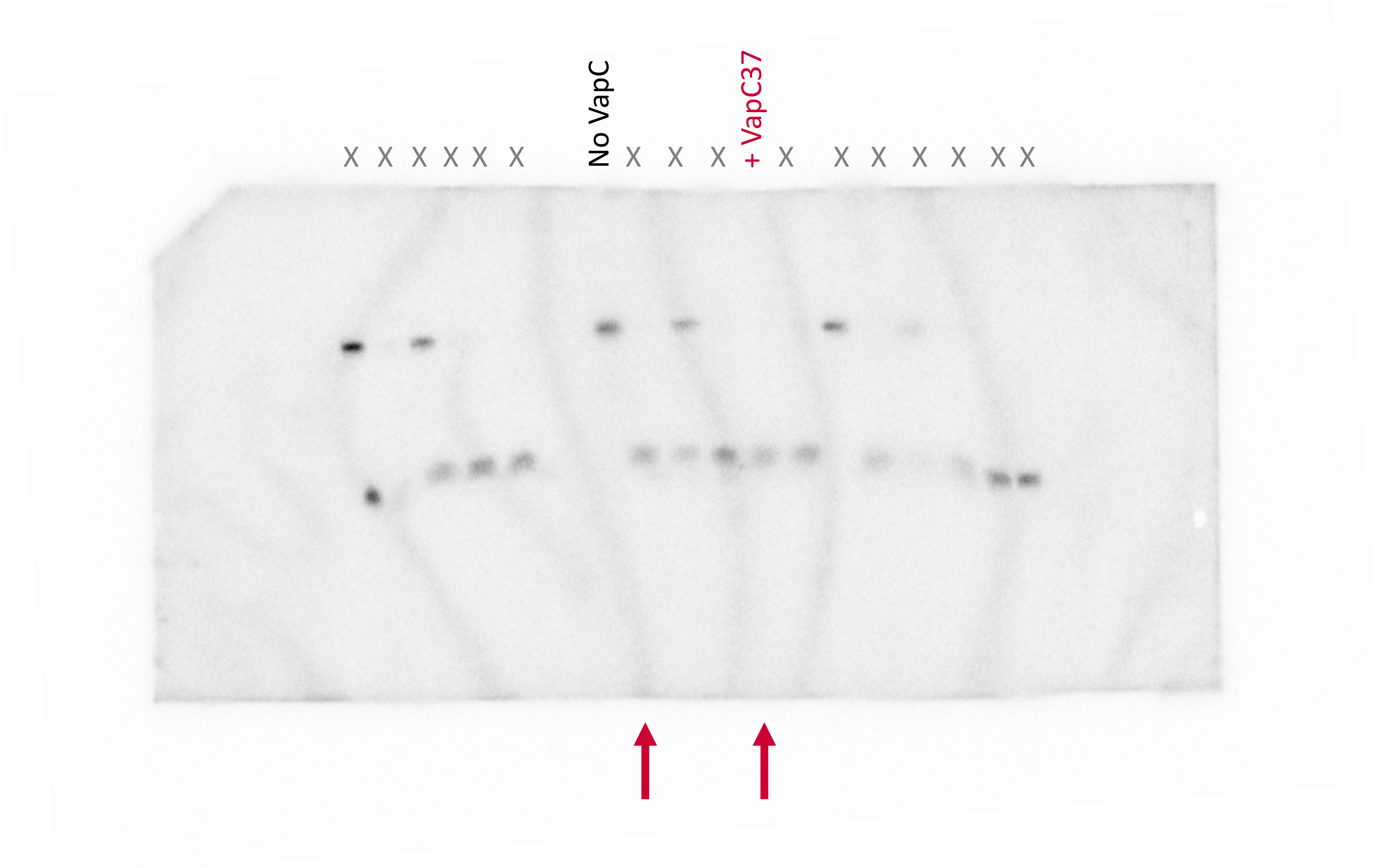

Figure 2F

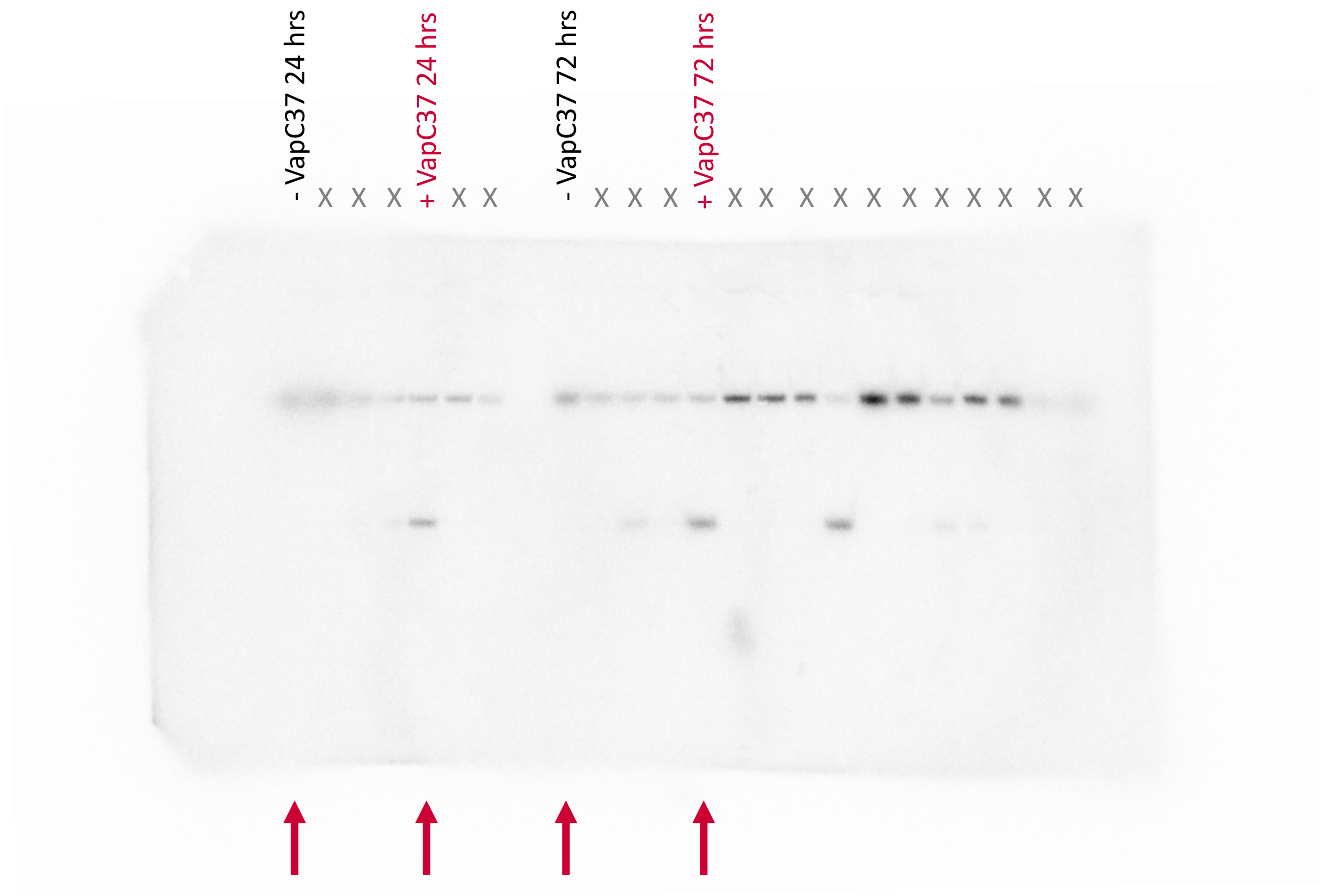
- VapC37 24 hrs
X X X
+ VapC37 24 hrs
X X
- VapC37 72 hrs
X X X
+ VapC37 72 hrs
X X X X X X X X X X X X

Supplemental Figure 1

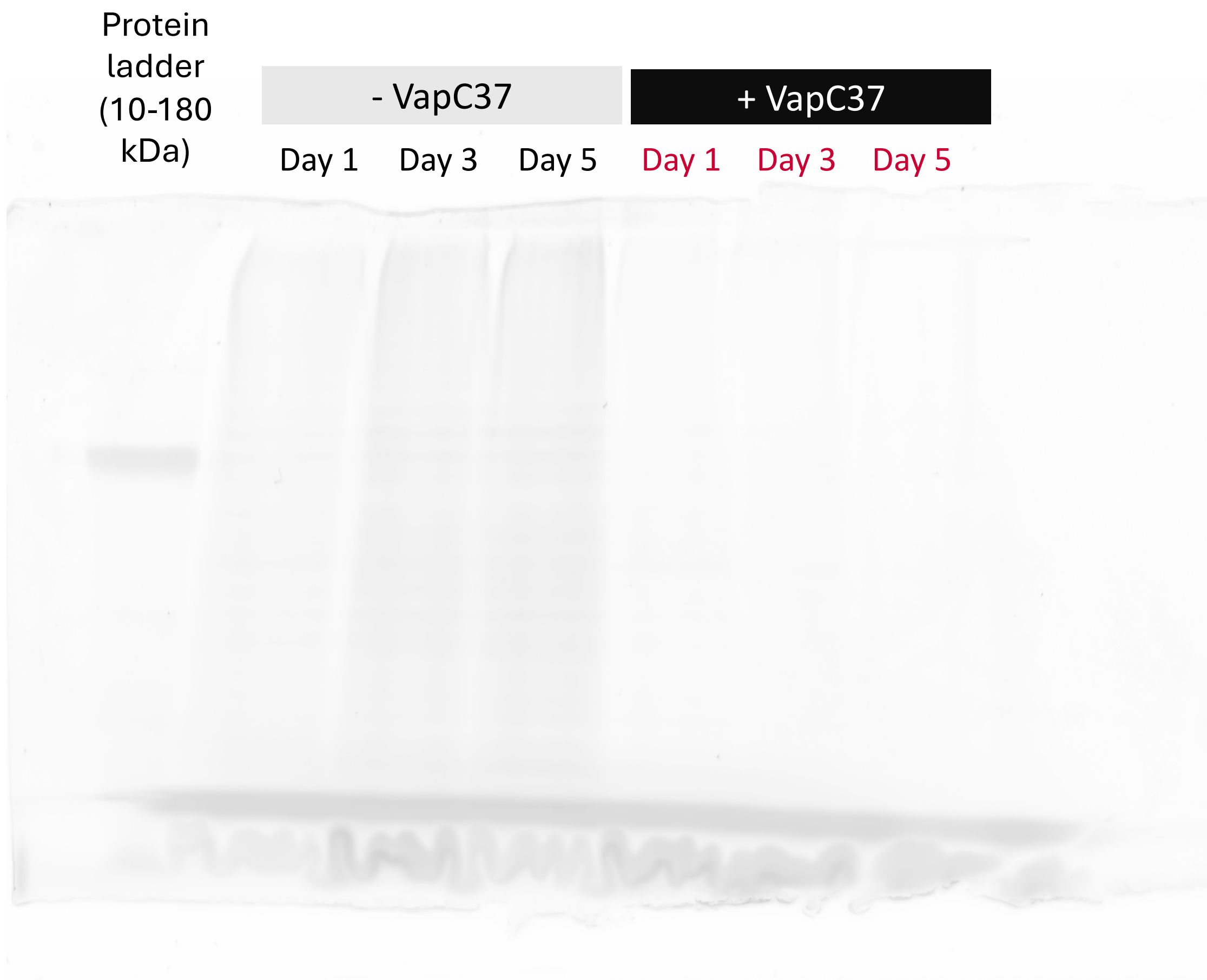

Supplement: Supplementary file 1 [file ppat.1014679.s003.pdf]
